# Genomic Insights into Cobweb Disease Resistance in *Agaricus bisporus*: A Comparative Analysis of Resistant and Susceptible Strains

**DOI:** 10.3390/jof11030200

**Published:** 2025-03-04

**Authors:** Guohui Cheng, Xiaoya An, Yueting Dai, Changtian Li, Yu Li

**Affiliations:** 1 Department of Plant Protection, Shenyang Agricultural University, Shenyang 110866, China; chengguohui2016@163.com; 2 Engineering Research Center of Chinese Ministry of Education for Edible and Medicinal Fungi, Jilin Agricultural University, Changchun 130118, China; anxiaoya2016@163.com (X.A.); daiyueting18@163.com (Y.D.)

**Keywords:** button mushroom, resistance determination, CAZymes, CYPs, NI-siderophore, fatty acid metabolism, pan-genome

## Abstract

*Agaricus bisporus*, a globally cultivated edible fungus, faces significant challenges from fungal diseases like cobweb disease caused by *Cladobotryum mycophilum*, which severely impacts yield. This study aimed to explore the genetic basis of disease resistance in *A. bisporus* by comparing the genomes of a susceptible strain (AB7) and a resistant strain (AB58). Whole-genome sequencing of AB7 was performed using PacBio Sequel SMRT technology, and comparative genomic analyses were conducted alongside AB58 and other fungal hosts of *C. mycophilum*. Comparative genomic analyses revealed distinct resistance features in AB58, including enriched regulatory elements, specific deletions in AB7 affecting carbohydrate-active enzymes (CAZymes), and unique cytochrome P450 (CYP) profiles. Notably, AB58 harbored more cytochrome P450 genes related to fatty acid metabolism and unique NI-siderophore synthetase genes, contributing to its enhanced environmental adaptability and disease resistance. Pan-genome analysis highlighted significant genetic diversity, with strain-specific genes enriched in pathways like aflatoxin biosynthesis and ether lipid metabolism, suggesting distinct evolutionary adaptations. These findings provide valuable insights into the genetic basis underlying disease resistance in *A. bisporus*, offering a foundation for future breeding strategies to improve fungal crop resilience.

## 1. Introduction

*Agaricus bisporus*, also known as the button mushroom, is one of the most economically significant edible fungi, widely cultivated for its nutritional and medicinal value, as well as its culinary appeal [1,2]. As a staple in global agriculture, it contributes significantly to food security and rural economies. However, the cultivation of *A. bisporus* faces challenges from fungal diseases such as dry bubble (caused by *Lecanicillium fungicola*) [3], wet bubble (caused by *Hypomyces perniciosus*) [4], and cobweb disease (caused by *Cladobotryum mycophilum*) [5,6], which result in the decrease in production. Among these, *C. mycophilum* is particularly notable because it can cause diseases in more than 13 mushroom species, including *A. bisporus*, *Pleurotus ostreatus*, *Lentinula edodes*, and *Morchella sextelata* [7]. Therefore, breeding highly resistant new germplasm has always been one of the vital objectives. *Agaricus bisporus* strains display varying degrees of resistance to specific diseases [6,8]. This trait can be differentiated among offspring [9,10], demonstrating the heritable nature of disease resistance in *A. bisporus* strains. Such heritability may be associated with the genetic background of the strains, gene expression patterns, and their intrinsic defense mechanisms [11,12]. While conventional breeding approaches have been hindered by the pseudohomothallic life cycle of *A. bisporus*, emerging genomic technologies offer new opportunities [13].

Whole-genome sequencing (WGS) and comparative genomics have revolutionized the field of fungi, particularly for trait-related gene discovery and molecular breeding [14,15], by providing comprehensive genomic resources that are essential for the identification of key genes and regulatory networks involved in pathogen defense, which include resistance genes, cytochrome P450 enzymes, and chitinases [16,17]. These approaches have enabled the identification of key genes associated with disease resistance, yield improvement, and environmental adaptation, paving the way for precision breeding strategies [18,19,20]. Comparative genomics, in particular, has provided valuable insights into the evolution of resistance across different fungal lineages [21,22,23,24]. For example, studies on *A. bisporus* resistance to *L. fungicola* had revealed a complex polygenic inheritance pattern, emphasizing the dual contribution of wild and cultivated alleles to disease resistance [9,10]. Similarly, research on *A. bisporus* resistance to *H. perniciosus* had identified key candidate genes involved in wet bubble disease resistance breeding [4]. Despite these advancements, significant gaps remain in our understanding. While several chromosome-level genomes of *A. bisporus* are available in the NCBI database, there is still a lack of strain-specific reference genomes, particularly for resistant and susceptible strains [25]. This limitation is especially pronounced in the context of *A. bisporus* resistance to cobweb disease, which is caused by *C. mycophilum*. Strain-specific reference genomes are critical for comparative analysis of disease resistance traits and for developing genetic markers to support marker-assisted breeding in *A. bisporus*.

Despite preliminary investigations into resistance-associated genomic features [10,17,26], systematic comparisons of resistant and susceptible strains remain critically lacking. The genomic information of *A. bisporus* regarding ecological adaptability, disease resistance, and nutritional component regulation is still incomplete, and further research and analysis are urgently needed. In this study, we performed whole-genome sequencing of a highly susceptible *A. bisporus* strain infected by *C. mycophilum* and conducted a comparative study with published genomic data from various strains of *A. bisporus* and other hosts of *C. mycophilum*, including *P. ostreatus*, *L. edodes,* and *M. sextelata*. The aim was to analyze the unique genetic variations or potential structural characteristics and explore the disease resistance-related genes, with the expectation of laying a molecular foundation for the disease resistance breeding of *A. bisporus*.

## 2. Materials and Methods

### 2.1. Resistance of Agaricus bisporus Basidiomes to Cobweb Disease

For the study of the resistance of *A. bisporus* basidiomes to cobweb disease, two mushroom strains of *A. bisporus* AB7 and AB58 were obtained from the Engineering Research Center of the Chinese Ministry of Education for Edible and Medicinal Fungi, Jilin Agricultural University, Changchun, China (Table S1). The pathogenic strain of *Cladobotryum mycophilum* CM30 (CMIDR1), an agent of cobweb disease, was used to test the resistance of *A. bisporus* basidiomes [6]. A 10 µL suspension of the pathogen CM30 with a concentration of 1 × 10^5^ spore/µL containing 0.05% tween-20 was inoculated onto the surface of *A. bisporus* basidiomes with a diameter of 9–14 mm [4]. Control treatment was carried out with sterile distilled water instead of suspension. For each strain (AB7 and AB58), six biological replicates were included per treatment, and all samples were cultivated under the same conditions in the Mushroom Base of Jilin Agricultural University. The characteristics of mushroom disease, such as lesion size, color, and depth, were measured at 96 h post-inoculation (hpi).

### 2.2. DNA Extraction, Genome Sequencing, and Assembly

First, the monokaryon strain AB7.31 was isolated from AB7 using the method described by Sun [27]. After culturing the strain AB7.31 on potato dextrose agar (PDA) medium at 25 °C for two weeks, its genomic DNA was extracted with the GP1 extraction technique. For further research, the identity of the fungus was confirmed through morphological characteristics, PCR amplification, sequencing of the ITS gene, and a BLAST search (https://blast.ncbi.nlm.nih.gov/Blast.cgi, accessed on 8 January 2025) on the GenBank database (Figure S1C). The quality of DNA was assessed by 1% agarose gel electrophoresis (Figure S1A) and 0.8% pulsed-field agarose gel electrophoresis (Figure S1B). The DNA was quantified using a Nanodrop 1000 (NanoDrop Technologies, Inc., Wilmington, DE, USA) and a Qubit 2.0 fluorometer (Thermo Fisher Scientific, Waltham, MA, USA) (Table S2). Next, sequencing DNA libraries were generated using NEBNext^®^ Ultra™ DNA Library Prep Kit for Illumina (New England Biolabs, Inc., Ipswich, MA, USA) according to the manufacturer’s protocol. Among these libraries, the one with an insert size of 350 bp was sequenced on the Illumina PE150 platform. Subsequently, the resultant sequencing data (2.57 Gb) were used for survey analysis and correcting the third-generation sequencing data (Table S3). A BluePippin device (Sage Science, Inc., Beverly, MA, USA) was used to select fragments larger than 20 kb for the SMRT Bell Template library. The quality and quantity of the constructed library were evaluated using a Qubit 2.0 fluorometer and an Agilent 2100 Bioanalyzer (Agilent Technologies, Santa Clara, CA, USA). Then, the whole genome of AB7 was sequenced by the Beijing Novogene Bioinformatics Technology Co., Ltd. (Novogene, Beijing, China) on the PacBio Sequel II platform. A total of 8.59 Gb HiFi reads (High Fidelity reads) were generated from subreads through the CCS (circular consensus sequence) algorithm (standard: min-passes = 3, min-rq = 0.99) [28]. These HiFi reads were assembled with Hifiasm v0.14.2-r315 software. Finally, the integrity of the genome assembly was assessed using BUSCO v3 (Benchmarking Universal Single-Copy Orthologs) [29,30].

### 2.3. Gene Prediction and Annotation

The assembled genome was annotated using a combination of de novo, homologous, and transcript-based prediction strategies. Specifically, this included the following: (A) Ab initio gene finding using a selection of the following software tools: GeneMarkHMM v4.32 [31], FGENESH v3.12 [32], Augustus v3.3.3 [33], SNAP v2016-07-28 [34], and GlimmerHMM v3.0.4 [31]. (B) Protein homology detection and intron resolution using the GeneWise v2.4.1 [35] software and the uniref90 non-redundant protein database (referring to *A. bisporus* var. *bisporus* H97 and *A. bisporus* var. *burnettii* H119_p4). (C) Alignment of known ESTs (Expressed Sequence Tags), full-length cDNAs, and most recently, Trinity [36] RNA-Seq assemblies to the genome. (D) PASA (Program to Assemble Spliced Alignments) [37] alignment assemblies based on overlapping transcript alignments from step (C). (E) Use of EVidenceModeler (EVM) [38] to compute weighted consensus gene structure annotations based on the above (A, B, C, D). (F) Use of PASA to update the EVM consensus predictions, adding UTR (Untranslated Regions) annotations and models for alternatively spliced isoforms (leveraging D and E). For the analysis of genome components, the interspersed repetitive sequences were predicted using RepeatModeler2 v2.0.1 [39] and RepeatMasker v4.1.2 [40]. Tandem Repeats (TRs) were analyzed by the MIcroSAtellite identification tool v2.1 [41] and TRF v4.09 (Tandem Repeats Finder) [42]. Transfer RNA (tRNA) genes were predicted using tRNAscan-SE v1.3.1 [43]. Ribosome RNA (rRNA) genes were analyzed using Barrnap v0.9 [44]. MicroRNA (miRNA), small nucleolar RNA (snoRNA), and small nuclear RNA (snRNA) were predicted by BLAST against the Rfam v14.9 [45,46] database. Finally, a whole genome BLAST search (e-value ≤ 1 × 10^−5^, identity ≥ 40%, coverage ≥ 40%) was performed against the following public databases: NR [47] (Non-Redundant Protein Database), Swiss-Prot [48], Pfam (Protein Families Database), KEGG [49] (Kyoto Encyclopedia of Genes and Genomes), GO [50] (Gene Ontology), and KOG [51] (Clusters of Orthologous Groups for Eukaryotic).

### 2.4. Analyses of CAZymes, Cytochrome P450s, and NI-Siderophore Synthetase Genes

The button mushroom (*A. bisporus*) is one of the hosts of *C. mycophilum*, which is reported to cause cobweb disease in many edible fungi, including *Pleurotus ostreatus*, *Lentinus edodes*, and *Morchella sextelata* [7,52]. To analyze the variation in CAZyme families in the different hosts of *C. mycophilum*, the protein-coding genes of the ten fungal genomes were annotated by the CAZy Database (Carbohydrate-Active enZYmes Database) [53]. Then, the GHs (Glycoside Hydrolases) families of these ten fungal genomes were clustered using the pheatmap package in R v4.4.2. Cytochrome P450s (CYPs) are a large family of heme-based proteins that catalyze the oxidation of many substrates. For comparative analysis, BLAST searches were performed on the ten fungal genomes from four different genera against the Fungal Cytochrome P450 database [54]. Subsequently, all CYPs from the ten strains were clustered using the R pheatmap package. Additionally, a line graph was generated to highlight the differences in CYPs between the strains AB7 and AB58. To analyze variation in the secondary metabolism (SM), we enriched and assessed the identity of SM biosynthetic gene clusters in the ten host genomes based on the antiSMASH database using the fungiSMASH option [55]. The secondary-metabolism gene clusters in these ten fungal genomes were then counted according to their types. A TBtool-II [56] was employed to obtain chromosomes and contigs information of the genomes. MapChart 2.32 [57] was used to visualize the chromosome and contig distributions of NI-siderophore genes clusters in the ten fungal genomes, based on the starting position of the genes and the chromosomal lengths. Subsequently, the characteristics of NI-siderophore gene clusters were compared. The prediction of conserved motifs of the NI-siderophore synthetase genes was carried out using the MEME [58], employing the default settings. Concurrently, the secondary and tertiary structures of the genes were predicted with the utilization of PredictProtein [59] and SWISS-MODEL [60], respectively. Finally, the subcellular localization of proteins encoded by NI-siderophore synthetase genes in different organisms was predicted using Cell-PLoc 2.0 [61].

### 2.5. Analyses of Potential Resistance Genes and Transport Factors

To further investigate the resistance differences among *A. bisporus* strains, BLAST searches were performed on the AB7 and AB58 genomes against the Plant TFDB [62] and PRGdb databases [63]. All the predicted resistance genes were visualized using a line graph, and cluster analysis was performed based on the domain characteristics of these resistance genes. All the predicted transcription factor families were analyzed using a line graph.

### 2.6. Collinearity Analysis of Agaricus bisporus Strains

Genomic alignment between the AB7 and AB58 genomes was conducted using the MUMmer v.3.23 [64] and LASTZ v.1.03.54 [65] tools. Comparative analysis identified syntenic blocks, which were filtered based on reliability criteria, considering sequence quality and syntenic accuracy factors. Genomic synteny was then analyzed using processed alignment data and selected blocks.

### 2.7. Pan-Genome Analysis of Agaricus bisporus Strains

Core and specific genes were analyzed using the CD-HIT v.4.6.1 [66] software for rapid protein clustering, with a threshold of 50% pairwise identity and a 0.7 length-difference cutoff in amino acids. Subsequently, a Venn diagram was drawn to illustrate the relationships among the samples, followed by KEGG enrichment analysis of strain-specific genes, with the top 20 pathways visualized.

### 2.8. Data Availability

The ITS sequence of *Agaricus bisporus* strain AB7 is available in the National Center for Biotechnology Information (NCBI) GenBank under the accession number PQ864803. This Whole Genome Shotgun project has been deposited at GenBank under the accession JBLEQQ000000000. The genomes used in this study include those of four *A. bisporus* strains, two *P. ostreatus* strains, two *L. edodes* strains, and one *M. sextelata* strain. These genomes can be accessed from the NCBI database at the following links (accessed on 10 January 2025), respectively:

*A. bisporus*: https://www.ncbi.nlm.nih.gov/datasets/genome/?taxon=5341

*P. ostreatus*: https://www.ncbi.nlm.nih.gov/datasets/genome/?taxon=5322

*L. edodes*: https://www.ncbi.nlm.nih.gov/datasets/genome/?taxon=5353

*M. sextelata*: https://www.ncbi.nlm.nih.gov/datasets/genome/?taxon=1174677

The software and databases used in this study can be downloaded and accessed in Table S24.

## 3. Results

### 3.1. Resistance Determination of Agaricus bisporus Strains to Cobweb Disease

**Figure 1:**
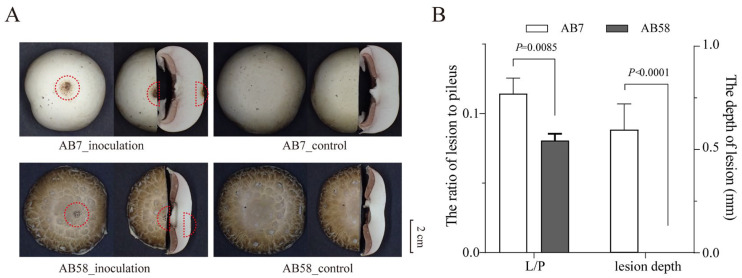
Resistance of *Agaricus bisporus* strains AB7 and AB58 to *Cladobotryum mycophilum* CM30. (**A**) Symptoms of cobweb disease caused by *C. mycophilum* on *A. bisporus* basidiomes at 96 h post-inoculation (hpi). The red dotted line indicates the inoculation site on the basidiomes. Scale bar = 1 cm. (**B**) Bar chart showing the severity of cobweb disease on the basidiomes. Results are expressed as the mean ± SD (n = 6). Statistical significance of the ratio of lesion area to pileus area (L/P) and lesion depth was determined using an independent two-sample *t*-test.

Artificially inoculating *Cladobotryum mycophilum* CM30 onto the surface of mushroom basidiomes induced typical brown pitting lesions on strain AB7 and AB58 at 96 hpi (Figure 1A). Basidiomes treated with sterile distilled water as controls remained asymptomatic. The lesion of the strain AB7 was darker than that of AB58. Furthermore, the longitudinal section of the basidiomes showed that AB7 tissue had suffered obvious damage. Both the lesion depth and L/P (the ratio of lesion to pileus) of AB7 were significantly greater than those of AB58 (Figure 1B), indicating that AB58 was more resistant to the infection of *C. mycophilum*.

### 3.2. Comparison of Genomic Features of Agaricus bisporus Strains AB7 and AB58

**Figure 2:**
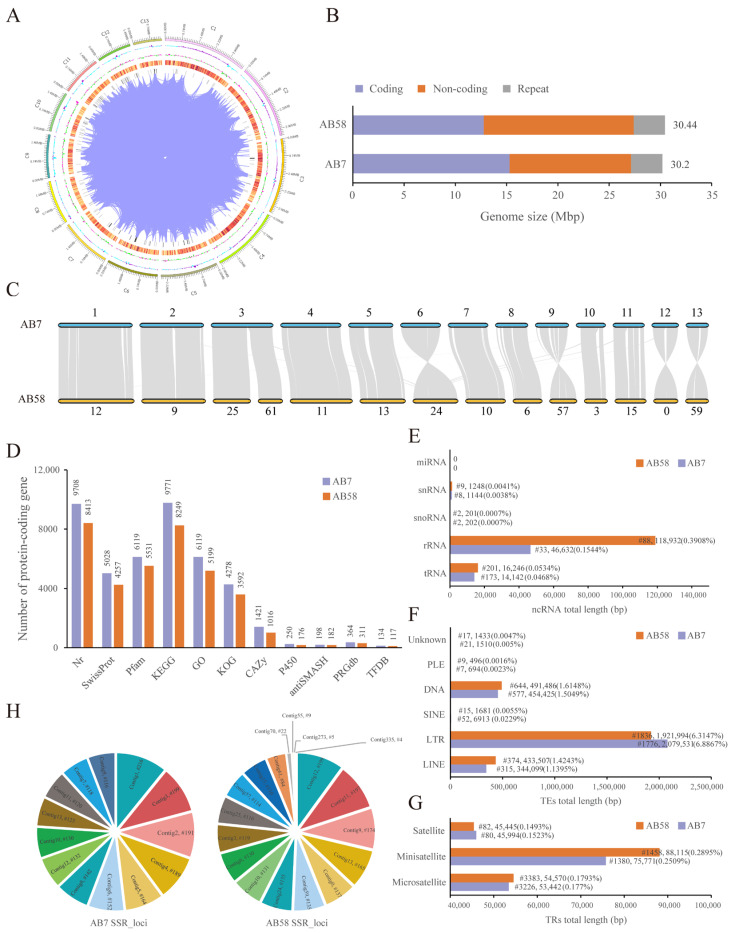
Characteristics of *Agaricus bisporus* strain AB7 and AB58 genomes. (**A**) Global view of *A. bisporus* strain AB7 genome. The outermost circle depicts 13 contigs of the AB7 genome. Moving inward, circle 2 shows the genomic GC content. The inner blue area indicates a lower GC content in this region compared to the genome-wide average, while the outer purple area indicates the opposite. The height of the peaks represents deviations from the average GC content. Circle 3 represents the genomic GC skew value. The inner green area indicates a lower G content than C in this region, and the outer pink area indicates the reverse. Circle 4 represents the gene density, with darker color corresponding to a higher gene density within the window. The middle circle represents chromosome duplication. (**B**) Genome sizes of strains AB7 and AB58. (**C**) Genomic collinearity between the AB7 and AB58 genomes. The numbers refer to the contigs. (**D**) Statistics of protein-coding genes annotated in 11 databases for the AB7 and AB58 genomes. (**E**) Statistics of non-coding RNAs (ncRNAs) across the AB7 and AB58 genomes. (**F**) Statistics of transposable elements (TEs) in the AB7 and AB58 genomes. (**G**) Statistics of tandem repeats (TRs) in the AB7 and AB58 genomes. (**H**) Distribution of simple sequence repeat (SSR) loci in the AB7 and AB58 genomes.

A total of 541,971 HiFi reads, representing 8.59 Gb of sequence data at 285 X coverage with a GC content of 46.52% (Table 1; Figure S3; Table S5), were generated from the genome of *A. bisporus* AB7 sequenced using the PacBio Sequel platform. The heterozygosity rate of the AB7 genome was determined to be 0.03% using 15-mer analysis (Figure S2; Table S4). The de novo assembly of the AB7 genome resulted in ~30.20 Mb, comprising 13 contigs with an N50 length of 2.49 Mb and average exon length of 284.6 bp (Table 1). BUSCO assessment results showed that 94.5% (716 out of 758 genes) of the genes were covered, indicating that the assembled genome of AB7 was of a high quality (Table S6). The Assembly of the *A. bisporus* AB7 genome was visualized using Circos v0.69 [67] software (Figure 2A).

The genome size of AB7 (30.20 Mb) was slightly smaller than that of AB58 (30.44 Mb), due to the large proportion of non-coding sequences in AB58 (Figure 2B; Table S7). Protein-coding sequences were predicted to account for approximately 50.63% and 41.93% of the AB7 and AB58 genomes, respectively. Furthermore, 10,218 genes of the AB7 were annotated by the 11 databases (Figure 2D; Table S12). Specifically, 9708 (95.01%) genes were annotated by Nr, 5028 (49.21%) by SwissProt, 6119 (59.88%) by Pfam, 9771 (95.63%) by KEGG, 6119 (59.88%) by GO, 4278 (41.87%) by KOG, 1421 (13.91%) by CAZy, 250 (2.45%) by P450, 198 (1.94%) by antiSMASH, 364 (3.56%) by PRGdb, and 134 (1.31%) by PlantTFDB, respectively (Figure S4). However, 8562 genes of the AB58 were annotated by the above databases.

For non-coding RNAs, 62,612 bp (0.21%) and 136,627 bp (0.45%) were predicted in the AB7 and AB58 genomes, respectively (Table 1), including 173 and 201 of tRNAs, 33 and 88 of rRNAs, 2 and 2 of snoRNAs, 8 and 9 of snRNAs, and no miRNA (Figure 2E; Table S8). Repetitive sequences accounted for approximately 10.14% and 9.98% of the AB7 and AB58 genomes, respectively (Table 1). Among them, the transposable elements (TEs) sequence length of the AB7 and AB58 genomes were 2.89 Mb (9.56%) and 2.85 Mb (9.37%), respectively, including 315 and 374 of long interspersed nuclear elements (LINEs), 1776 and 1836 of long terminal repeats (LTRs), 52 and 15 of short interspersed nuclear elements (SINEs), and 577 and 644 of DNA transposons (Figure 2F; Table S9). The tandem repeats (TRs) sequence length of the AB7 and AB58 genomes were 0.18 Mb (0.58%) and 0.19 Mb (0.61%), respectively (Figure 2G; Table S10). A total of 1960 and 1965 simple sequence repeats (SSR) loci were predicted for the AB7 and AB58 genomes, respectively (Table S11). The distribution of these SSR loci in the genomes was illustrated in Figure 2H.

### 3.3. Collinearity Analysis of Agaricus bisporus Strains AB7 and AB58

The whole-genome collinearity analysis was conducted for strains AB7 and AB58 in relation to the cobweb disease caused by *C. mycophilum*. The syntenic genes among *A. bisporus* strains were explored through collinearity analysis using MUMmer v3.23 and LASTZ v1.03.54 (Figure 2C). According to the collinearity results, 13 contigs of AB7 were highly conserved syntenic blocks shared with AB58. Among these contigs, four contigs showed inversions between AB7 and AB58, including contig6 of AB7 being inverted with contig24 of AB58, contig9 being inverted with contig57, contig12 being inverted with contig0, and contig13 being inverted relative to contig59. In addition, contig3 of AB7 formed a conserved syntenic block with contig25 and contig61 of AB58, suggesting that AB7 and AB58 have adapted to various survival conditions.

### 3.4. Carbohydrate-Active enZymes (CAZymes) Analysis

**Figure 3:**
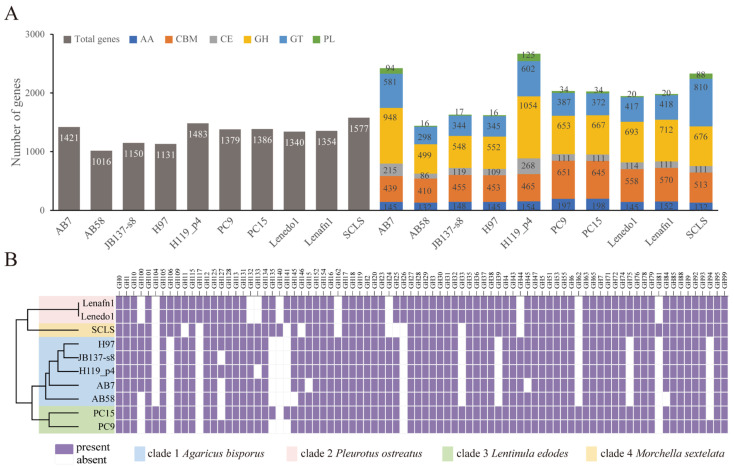
Comparison of carbohydrate-active enzymes (CAZymes) across ten hosts of the fungal pathogen *Cladobotryum mycophilum*. (**A**) Statistics on the total number of genes and a comparison of CAZyme families predicted in the genomes of the ten hosts. The gene families are referred to by their standard abbreviations: AA (Auxiliary activities), CBM (Carbohydrate-binding modules), CE (Carbohydrate esterases), GH (Glycoside hydrolases), GT (Glycosyl transferases), and PL (Polysaccharide lyases). (**B**) Binary heatmap showing the distribution of 84 Glycoside Hydrolase (GH) classes across the genomes of the ten hosts. Gene counts are normalized to a 0–1 scale (by CAZyme class) for visualization. Purple boxes indicate the presence of genes, while white boxes indicate their absence.

To investigate the role of carbohydrate processing, we annotated and assessed the identity of CAZymes in the ten host genomes (Figure 3). More CAZyme families were identified in strain H119_p4, with 1483 genes annotated (1054 for glycoside hydrolases (GH) family) (Figure 3A; Table S13). Strain AB7 had the second-largest number of predicted CAZyme families, with 1421 genes annotated (948 for GH family). In contrast, AB58 was predicted to contain 1441 genes annotated (499 for GH family) belonging to CAZyme families. It is worth noting that 27 of 84 GH classes varied obviously across the ten host genomes, suggesting the crucial importance of GH family variation (Figure 3B; Table S14). Based on the binary heat map of the GH classes, the ten strains were clustered into four clades. The result was completely consistent with the classification of the ten strains, implying a relationship between the GH family and fungal nutrition. For instance, the specific deletion of GH75, GH39, GH194, and GH135 in *A. bisporus* might explain why it is difficult to utilize galactose [68]. Specific GH classes are present in specific genera, such as GH26 and GH117 in *L. edodes*, GH62 in *P. ostreatus*, and GH81 in *M. sextelata*. In addition, as observed between strain AB7 and AB58, the specific deletion of GH15 and GH45 in strain AB7, which are involved in β-1,4-glucosidase activity, may result in reduced cellulose utilization [69].

### 3.5. Cytochrome P450 (CYPs) Family Analysis

**Figure 4:**
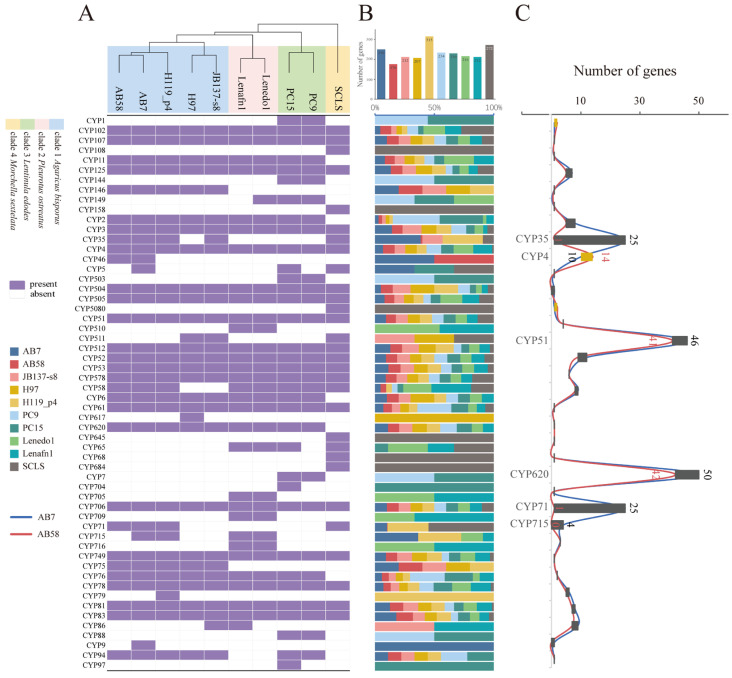
Analysis of Cytochrome P450 (CYPs) family across ten hosts of the fungal pathogen *Cladobotryum mycophilum*. (**A**) Binary heat map showing the distribution of 56 CYP classes across the genomes of the ten hosts. Gene counts are normalized to a 0–1 scale (by CYP class) for visualization. Purple boxes indicate the presence of genes, while white boxes indicate their absence. (**B**) Statistics on the total number of genes and the abundance of CYPs predicted in the genomes of the ten hosts. (**C**) Line plot illustrating the disparity in the number of genes predicted in 33 CYP classes between the AB7 and AB58 genomes. Blue line indicates the strain AB7, while red line indicates the strain AB58. The bar graphs quantify the difference in the number of these genes. Black bars indicate more genes annotated in AB7 than AB58 within a given class, while gold bars signify the opposite.

CYPs have emerged as a research focus for inhibiting pathogenic fungi and as a potential antifungal strategy. To gain insight into the functional genes of CYPs, we annotated and analyzed the identity of CYP families in the ten host genomes (Figure 4). In total, 56 different CYP classes were identified across the ten host genomes (Table S15). Among these, 38 of these CYP classes displayed patterns of differential abundance between the four clades (Figure 4A). Specifically, 17 CYP classes were uniquely presented in four genera: two classes (CYP75 and CYP146) in *A. bisporus*, four classes (CYP705, CYP510, CYP709, and CYP716) in *L. edodes*, five classes (CYP144, CYP1, CYP503, CYP7, and CYP88) in *P. ostreatus*, and six classes (CYP158, CYP108, CYP5080, CYP645, CYP68, and GH684) in *M. sextelata*. These differences are closely related to their metabolic functions and defense mechanisms. Although strain AB58 had the lowest number of CYP genes among the ten host genomes, the abundance of specific CYP classes, such as CYP75 and CYP146, remained relatively consistent with the other four genomes of *A. bisporus* (Figure 4B). Notably, there were significant differences in the number of genes across the six CYP classes between strain AB7 and AB58 (Figure 4C). More CYP35 genes (black bar) were identified in strain AB7 than in strain AB58, as were CYP51, CYP620, CYP71, and CYP715. In contrast, strain AB58 had a higher number of CYP4 genes (gold bar), which are involved in fatty acid metabolism, compared to strain AB7.

### 3.6. NI-Siderophore Synthetase Genes Analyses

**Figure 5:**
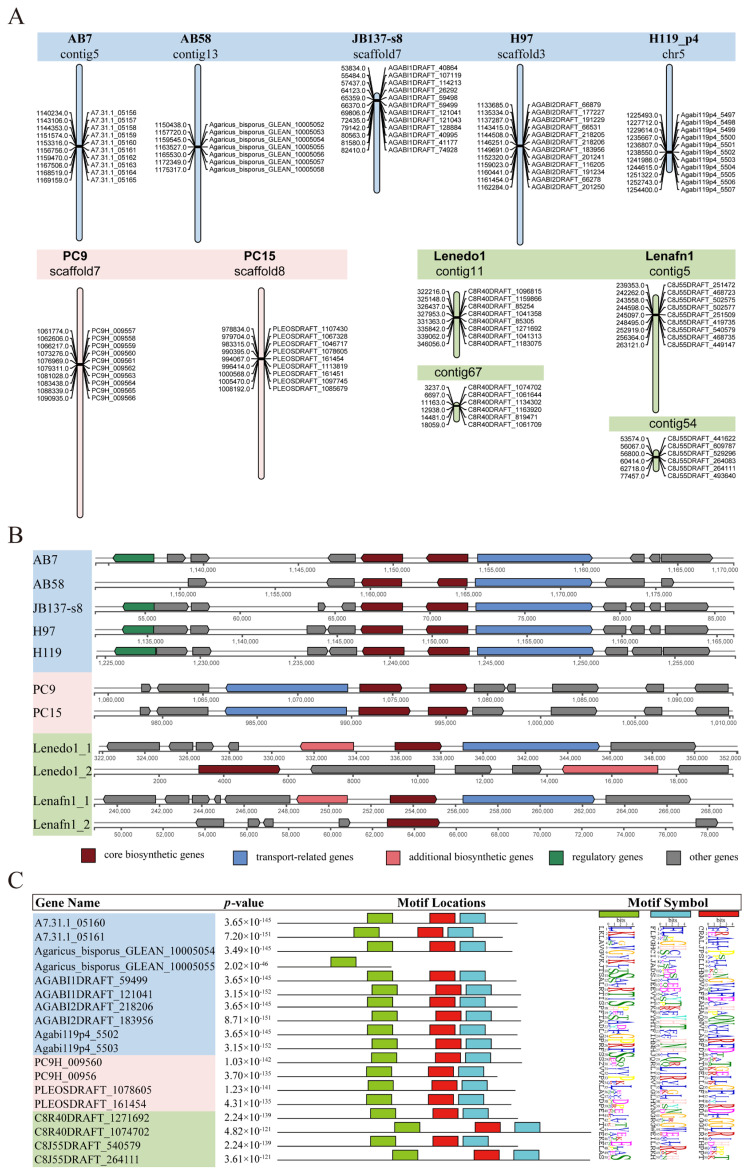
Analysis of NRPS-independent siderophore (NI-siderophore) synthetase genes across ten hosts of the fungal pathogen *Cladobotryum mycophilum*. (**A**) Distribution of 11 NI-siderophore synthetase gene clusters across 11 contigs in nine of the ten host genomes. (**B**) Comparison of structural, compositional, and organizational features of NI-siderophore synthetase-related gene clusters. (**C**) Motif analysis for NI-siderophore synthetase genes. Boxes of different colors represent distinct motif, highlighting conserved regions in the gene sequences.

To analyze the role of secondary metabolites (SM), we enriched and assessed the identity of SM biosynthetic gene clusters in the ten host genomes (Figure S5). In total, 21, 21, 22, 22, and 22 clusters were identified in *A. bisporus* strain AB7, AB58, JB137-s8, H97, and H119_p4, respectively (Table S16). Only 15 clusters were identified in *M. sextelata* strain SCLS genome. In contrast, 43, 42, 40, and 29 clusters were identified in the genomes of *P. ostreatus* strain PC9, PC15, and *L. edodes* strain Lenedo1, Lenafn1, respectively. Among these, 11 NI-siderophore synthetase gene clusters, involved in enhancing disease resistance, were identified in four *Agaricus* strains, two *P. ostreatus* strains, and two *L. edodes* strains (Figure 5; Table S17). These 11 gene clusters were distributed across 11 contigs of the nine genomes mentioned above, with each of the two *L. edodes* strains containing two NI-siderophore gene clusters (Figure 5A). In *L. edodes*, each of the four contigs contained a single core NI-siderophore biosynthetic gene, ranging from 2198 to 2511 bp. In contrast, two core NI-siderophore genes were present across seven contigs of five *A. bisporus* strains and two *P. ostreatus* strains, ranging from 1572 to 2717 bp (Figure 5B). Additionally, an extra biosynthetic gene was found in all *L. edodes* contigs, except contig54 of strain Lenafn1, while a regulatory gene of the NI-siderophore cluster was present in all *A. bisporus* contigs except contig13 of strain AB58. All core NI-siderophore genes were predicted to possess α-helix, β-fold, and random coil structures. Notably, a core biosynthetic gene (GLEAN_10005055) in AB58 with a length of 1572 bp, was significantly shorter than other core genes in *A. bisporus*, providing new insights into the resistance of *A. bisporus* to cobweb disease. Furthermore, gene GLEAN_10005055 in strain AB58 contains only one motif, whereas other NI-siderophore synthetase genes possess three motifs (Figure 5C).

Subcellular localization predictions indicated that the proteins encoded by NI-siderophore synthetase genes in AB58 exhibit more complex functionality, localizing to the extracellular space, cell membrane, cytoplasm, endoplasmic reticulum, and nucleus (Table S18). This suggests that these proteins may confer higher environmental adaptability and disease resistance in AB58 compared to other strains.

### 3.7. Analyses of Potential Resistance Genes and Transport Factors

**Figure 6:**
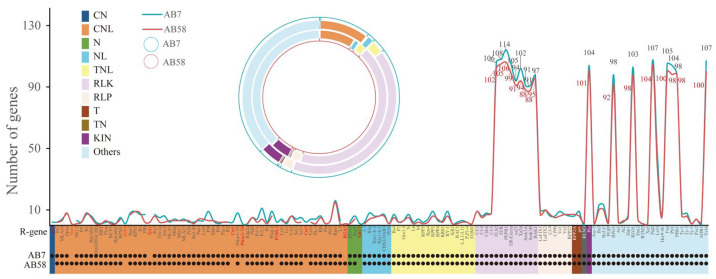
Analysis of resistance genes (R-genes) predicted in the *Agaricus bisporus* strains AB7 and AB58. In the concentric circles, the area enclosed by the blue circle depicts the R-gene classes predicted in strain AB7, while the area enclosed by the red circle represents the corresponding classes in strain AB58. Squares of various colors denote different classes of R-genes. Black dots indicate whether an R-gene is predicted to be present in both strains AB7 and AB58.

In order to further investigate the resistance disparities among *A. bisporus* strains, we have carried out the identification and analysis of potential resistance genes (R-genes) and transcription factors (TFs). A total of 364 genes were predicted to be classified into 11 classes in the AB7 genome, and 311 genes were predicted to be classified into 10 classes in the AB58 genome (Table S19). Among these, receptor-like kinase (RLK) class, which is involving in pathogen recognition through the activation of the mitogen-activated protein kinase (MAPK) signaling pathway, was abundant in the two genomes, accounting for 40.79% and 42.45% of the total resistance genes, respectively (Figure 6; Table S20). In AB58, seven genes (namely *pb1*, *RPP8*, *Cre1*, *Pikm2-TS*, *FOM-2*, *Cre3*, and *NRC1*) were predicted to be deleted, while in AB7, three genes (namely *Hero*, *XA1*, and *RGA5*) were predicted to be deleted. The deletion of these genes may contribute to the observed differences in resistance between the strains to certain pathogens. Meanwhile, a total of 15 TF families were predicted in strains AB7 and AB58, with the number of annotated genes being 137 and 117, respectively (Figure S6; Table S21). Specifically, 53 and 44 TFs of the C2H2 family, involved in post-transcriptional mRNA modification functions, were predicted in the strain AB7 and the strain AB58, respectively. Similarly, 21 and 15 TFs of the C3H family were predicted in AB7 and AB58, respectively. These differences may lead to different levels of refinement in gene expression regulation between the two strains, thereby affecting their growth and resistance capabilities.

### 3.8. Analysis on Strain-Specific Genes in the Pan-Genome of Strains AB7 and AB58

**Figure 7:**
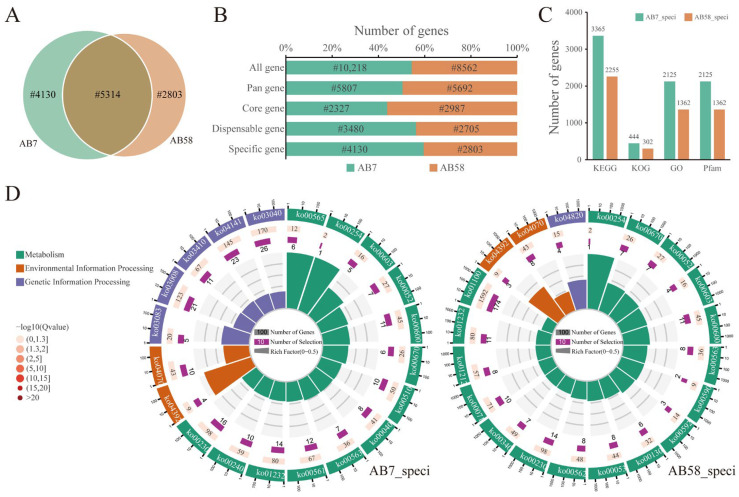
Analysis of the pan-genome of the *Agaricus bisporus* strains AB7 and AB58. (**A**) Venn diagram depicting the relationships among the samples. (**B**) Illustration of the genetic composition of the pan-genome. (**C**) Statistics on the functional annotation of strain-specific genes in AB7 and AB58. (**D**) KEGG clustering analysis of strain-specific genes in AB7 and AB58. The outermost circle represents the top 20 KEGG terms. The second circle shows the number of background genes and their Q-values in each pathway. The third circle indicates the number of genes, while the fourth circle represents the Rich Factor value for each pathway.

To explore the functions of strain-specific genes, we constructed a monokaryotic pan-genome of *A. bisporus* representing strains AB7 and AB58. A Venn diagram was created to illustrate the relationships between the samples (Figure 7A). In total, the pan-genome consisted of 11,499 gene families, among which 46.21% were core gene families and 53.78% were dispensable gene families. Of these, 4130 genes were specific to strain AB7, while 2803 genes were specific to strain AB58 (Figure 7B; Table S22). KEGG pathway analysis indicated that the strain-specific genes of AB7 and AB58 were significantly enriched in aflatoxin biosynthesis and the Hippo signaling pathway (Figure 7D; Table S23), which could be attributed to the similar domains of these specific genes. However, the strain-specific genes of AB7 were significantly enriched in ether lipid metabolism.

## 4. Discussion

In this study, artificial inoculation experiments were conducted using six biological replicates for each strain (AB7 and AB58). With a total of 24 samples, a *t*-test and variance analysis were performed. The experiments revealed that strain AB7, infected with cobweb disease caused by *Cladobotryum mycophilum*, exhibited darker lesions, greater lesion depth, and a higher lesion-to-pileus (L/P) ratio compared to AB58 (Figure 1). These findings confirm that AB58 possesses stronger resistance to cobweb disease, consistent with previous studies [6]. AB58, originating from the Tibetan Plateau wild population, has demonstrated not only higher genetic diversity but also remarkable resistance to *Hypomyces perniciosus*, unlike the more susceptible European and American populations [70]. This enhanced resistance may be attributed to specific genes or gene combinations involved in pathogen recognition, signal transduction, and defense substance synthesis [4,27,71]. To conduct comparisons and analyses at the whole-genome level, HiFi sequencing of *Agaricus bisporus* strain AB7 was performed using the PacBio Sequel II SMRT sequencing technology (Novogene, Beijing, China), generating a high-quality genome (Figure 2). The genome size of strain AB7 (30.20 Mb) is slightly smaller than that of AB58 (30.44 Mb). This difference is primarily attributed to the higher proportion of non-coding sequences in the genome of strain AB58 and its complex wild genetic background [27]. A larger genome might provide more genetic information for the strain to cope with intricate environmental challenges, yet it could also be associated with higher energy consumption [72,73]. Non-coding sequences play a crucial role in regulating gene expression [74]. Specifically, small RNAs (e.g., siRNAs and miRNAs) and long non-coding RNAs (lncRNAs) are vital for pathogen resistance, as they modulate immune-related gene expression and silence foreign genetic elements, including viral RNAs and transposons [74]. These regulatory mechanisms enable hosts to mount robust defense responses against pathogens, thereby enhancing disease resistance. Given that more non-coding sequences in strain AB58 were annotated compared to strain AB7, this implies that AB58 possesses a more elaborate gene expression regulatory network, which may contribute to its higher disease resistance. Additionally, the proportion of protein-coding sequences in strain AB7 is higher than that in strain AB58, representing a significant genomic disparity between the two strains. This disparity may potentially influence strain-specific characteristics such as adaptability, pigmentation, and disease resistance [75,76,77]. While HiFi sequencing and complementary strategies significantly improved assembly quality, some gaps and potential errors remain. Specifically, the results of strain-specific genes or inversions detected in the bioinformatics analysis still require experimental validation. For instance, fluorescence in situ hybridization (FISH) could be employed to verify inversions at the cytological level.

CAZymes play a pivotal role in fungal carbohydrate metabolism. A comprehensive comparison and evaluation of CAZymes across the ten hosts of *C. mycophilum* helps us explore nutrient utilization in each host meticulously (Figure 3). Among the ten hosts, 27 glycoside hydrolase (GH) classes exhibited significant differences, highlighting the importance of GH family variation in fungal carbohydrate processing [73]. Specific GH classes in different genera are linked to particular degradation functions. For instance, GH26 and GH117 in *Lentinula edodes*, GH62 in *Pleurotus ostreatus*, and GH81 in *Morchella sextelata* are closely associated with xylan or plant biomass degradation. These findings imply that different fungal genera have evolved specific CAZymes to efficiently break down the substrates available in their habitats [78,79]. Specifically, the deletion of specific GH15 and GH45 in AB7 may result in a diminished ability to utilize cellulose. As cellulose is a major component of plant biomass, the capacity to degrade it is essential for fungi that depend on plant-derived substrates [80]. The absence of these specific GH classes in AB7 may restrict its access to certain carbon sources, thus, affecting its competitiveness in the natural environment [81]. According to the analysis of CYP families across 10 hosts of the pathogen above (Figure 4), 38 out of 56 identified CYP classes show differential abundance patterns among the four genera. This indicates the high diversity of CYPs in fungi and their potential roles in strain-specific functions. For example, two classes of CYP in *A. bisporus* are involved in fatty acid metabolism and cell structure maintenance; four classes in *L. edodes* are related to hormone regulation, detoxification, etc.; five classes in *P. ostreatus* are involved in functions such as pigment and fatty acid metabolism; and six classes in *M. sextelata* are associated with metabolism, spore-related processes, and environmental response [72]. Furthermore, compared with AB7, strain AB58 contains more CYP4-like genes related to fatty acid metabolism. This difference suggests a potential role for these genes in modulating the composition and fluidity of the cell membrane, which could influence physiological functions and environmental adaptability [8,79]. Specifically, the enrichment of the CYP4 subfamily in AB58 may contribute to membrane remodeling through the ω-hydroxylation of fatty acids, a process previously linked to defense responses against oxidative stress in other organisms [82,83]. However, while these observations are consistent with a potential role in stress adaptation, further experimental validation is required to establish a direct causal relationship between CYP4-like genes and cobweb disease resistance in *A. bisporus*.

Differences in SM biosynthetic gene clusters among the four genera suggest evolutionary adaptations to diverse ecological niches [79]. For example, the higher number of clusters in *P. ostreatus* and *L. edodes* might enable the synthesis of various secondary metabolites for nutrient acquisition or competitive defense, while fewer clusters in *M. sextelata* suggest alternative survival mechanisms (Figure 5). Siderophores, supplying essential iron for the growth of strains, are closely associated with the disease resistance of fungal pathogens [84,85]. Furthermore, the 11 identified NI-siderophore gene clusters involved in enhancing disease resistance were distributed across 11 contigs in three genera. Perhaps *M. sextelata* relies on other mechanisms to maintain survival and adapt to the environment. Although NI-siderophore synthetase gene clusters are predicted in each strain of *L. edodes*, only one core gene is distributed in an NI-siderophore gene cluster. As a result, the number of NI-siderophore synthetase genes in each individual strain is the same. Among these 18 NI-siderophore genes, the core gene GLEAN_10005055 in strain AB58 has unique length and motif characteristics, resulting in differences in the structure and function of the protein it encodes compared to others [84]. This provides new insights into the potential role of NI-siderophore genes in the resistance of *A. bisporus* to cobweb disease. By investigating the interaction network between this gene and other related genes, we can gain a deeper understanding of its potential contribution to disease resistance. Moreover, the complex subcellular localization of the proteins encoded by NI-siderophore synthetase genes in strain AB58 implies that they can function in multiple cellular regions and participate in different physiological processes. This multifunctionality may endow strain AB58 with stronger environmental adaptability and disease-resistance capabilities compared to other strains, enabling it to better cope with different environmental conditions and threats from pathogens [81]. However, these observations are based on genomic and structural analyses, and further functional validation is required to establish causal links between these genomic differences and resistance phenotypes.

The differences in the quantity and classification of potential resistance genes between the *A. bisporus* strain AB7 and AB58 genomes reflect that they may have faced different selection pressures during evolution, thus, developing distinct resistance strategies (Figure 6). The deletion of different genes in AB58 and AB7 may alter their defense capabilities against specific pathogens. For instance, the deletion of genes such as *Hero*, *XA1*, and *RGA5* genes in AB7 may make it more susceptible to pathogens that rely on these genes for recognition and defense. This observation is an entry point for further research on the specific roles of these genes in the disease-resistance mechanism of *A. bisporus*. The differences in the numbers of C2H2 and C3H family transcription factors imply that there may be differences in post-transcriptional mRNA modification and other related gene-expression regulation processes between the two strains. The functions of these transcription factors may require more research to fully understand their role in post-transcriptional mRNA expression regulation. Meanwhile, pan-genome analysis provides a comprehensive perspective on the genetic constitution of *A. bisporus* strain AB7 and AB58 (Figure 7). The high proportion of dispensable gene families in the *A. bisporus* pan-genome (53.78%) reflects a significantly high level of genetic diversity among the strains [77,86]. The variation in the number of strain-specific genes between AB7 and AB58 suggests that these two strains have followed distinct evolutionary trajectories. The enrichment of specific genes related to aflatoxin biosynthesis and the Hippo signaling pathway in both strains may highlight the importance of these pathways for their survival or competitive advantage. Aflatoxin biosynthesis could be linked to the production of secondary metabolites that serve defensive or competitive roles [87]. Notably, the additional enrichment of AB7-specific genes in ether lipid metabolism is particularly intriguing. Ether lipids, as essential components of cell membranes, are involved in various biological processes, including signal transduction [88]. This finding may indicate that AB7 has evolved a unique membrane-related adaptation mechanism to cope with specific environmental challenges, such as temperature fluctuations, pH variations, or exposure to toxins.

## 5. Conclusions

This study compared the genomes of *Agaricus bisporus* strains AB58 (resistant) and AB7 (susceptible) to cobweb disease caused by *Cladobotryum mycophilum*. AB7 exhibits a smaller genome with fewer non-coding sequences and lacks specific glycosyl hydrolases (GHs), which may reduce its cellulose utilization and environmental competitiveness. In contrast, AB58 contains more CYP4-like genes involved in fatty acid metabolism and unique NI-siderophore synthetase genes, which likely enhance its cell membrane function, environmental adaptability, and disease resistance. Notably, this study provides the first evidence of structural variation in NI-siderophore synthetase genes associated with cobweb disease resistance in basidiomycetes, offering a potential avenue for further investigation. Differences in resistance-related genes and transcription factors suggest distinct regulatory and defense strategies. Pan-genome analysis reveals high genetic diversity, with AB7 and AB58 following different evolutionary paths. AB7’s enrichment in ether lipid metabolism indicates a unique membrane-related adaptation mechanism. The Tibetan Plateau origin of AB58 may have been selected for enhanced stress-response mechanisms through extreme environmental pressures. In contrast, cultivated strains such as AB7 and H97 may have experienced a weakening of stress-response mechanisms during domestication. While these findings provide valuable insights into the genomic basis of cobweb disease resistance, further functional validation is required to establish causal links between the observed genetic differences and resistance phenotypes. Future work should include functional assays (e.g., CRISPR-based knockouts), transcriptomic studies, and metabolomic analyses to validate the roles of CYP4-like genes, NI-siderophore synthetase genes, and other candidate genes in disease resistance.

## Figures and Tables

**Table 1:** Comparison of the genomes of *Agaricus bisporus* strains AB7 and AB58.

Items	AB7	AB58
Genome size (Mb)	30.2	30.44
Coverage (×)	285	282
Contigs (#)	13	18
N50 length (Mb)	2.5	2.3
GC content (%)	46.52	46.53
Gene number (#)	10,218	8562
Average CDS length (bp)	1490.82	1490.55
Gene total length (bp)	15,288,553	12,762,102
Average exon number (#)	5.9	5.55
Average exon length (bp)	284.6	268.6
Repeat sequence (bp)	3,062,379 (10.14%)	3,038,727 (9.98%)
ncRNAs (bp)	62,120 (0.21%)	136,627 (0.45%)

## Data Availability

The data of *Agaricus bisporus* strain AB7 genome is available in GenBank under the accession number PQ864803 (ITS) and JBLEQQ000000000 (WGS).

## Supplementary Materials

The following supporting information can be downloaded at: https://www.mdpi.com/article/10.3390/jof11030200/s1, Table S1: A list of *Agaricus bisporus* strains in this study and their specific characteristics; Table S2: Assessment of DNA quality from the monokaryotic sample of *Agaricus bisporus* AB7; Table S3: Statistics for Illumina PE150 sequencing data of *Agaricus bisporus* AB7 genome; Table S4: Genomic survey of *Agaricus bisporus* strain AB7 using 15-mer based on Illumina PE150 sequencing data; Table S5: Statistics of HiFi reads for *Agaricus bisporus* AB7 genome generated from subreads through the circular consensus sequence (CCS) algorithm; Table S6: Statistics of BUSCO evaluation for *Agaricus bisporus* AB7 genome; Table S7: Overall comparison of the genomes of *Agaricus bisporus* strains AB7 and AB58; Table S8: Statistics of non-coding RNA within the genomes of *Agaricus bisporus* strains AB7 and AB58; Table S9: Statistics of TEs within the genomes of *Agaricus bisporus* strains AB7 and AB58; Table S10: Statistics of TRs within the genomes of *Agaricus bisporus* strains AB7 and AB58; Table S11: Statistics of SSRs within the genomes of *Agaricus bisporus* strains AB7 and AB58; Table S12: Protein-coding gene annotation in *Agaricus bisporus* strains AB7 and AB58; Table S13: Statistics of CAZyme families across ten genomes; Table S14: Distribution of 84 GH classes across ten genomes; Table S15: Distribution of 56 P450 classes across ten genomes; Table S16: Statistics of secondary metabolite gene clusters across ten genomes; Table S17: Characteristics of 11 NI-siderophore synthetase gene clusters across nine genomes; Table S18: Subcellular localization of proteins encoded by NI-siderophore synthetase genes; Table S19: Statistics of resistance genes identified in the PRGdb database within the genomes of *Agaricus bisporus* strains AB7 and AB58; Table S20: Distribution of resistance gene classes within the genomes of *Agaricus bisporus* strains AB7 and AB58; Table S21: Statistics of transport factors identified in the Plant TFDB database within the genomes of *Agaricus bisporus* strains AB7 and AB58; Table S22: The genetic composition of the pan-genome of strains AB7 and AB58; Table S23: Statistics on the functional annotation of strain-specific genes in AB7 and AB58; Table S24: The software and databases used in this study; Figure S1: Quality inspection of DNA from *Agaricus bisporus* strain AB7; Figure S2: 15-mer frequency distribution and GC-sequencing depth correlation analysis of *Agaricus bisporus* strain AB7 genome; Figure S3: HiFi read and gene length distributions of the *Agaricus bisporus* AB7 genome; Figure S4: Visualized functional annotation of the *Agaricus bisporus* strain AB7 using KEGG, KOG, Nr, and GO databases; Figure S5: Statistical analysis of the annotation results of ten genomes in the antiSMASH database; Figure S6: Statistical analysis of TF families in *Agaricus bisporus* strains AB7 and AB58 predicted by Plant TFDB.
