# Supplementary material for: Genomic Insights into Cobweb Disease Resistance in Agaricus bisporus: A Comparative Analysis of Resistant and Susceptible Strains
Source: J Fungi (Basel). 2025 Mar 4;11(3):200. doi: 10.3390/jof11030200 (PMC11942895; doi:10.3390/jof11030200)
Supplement: Supplementary file 1 [file jof-11-00200-s001.zip › jof-3492928-supplementary.pdf]

Genomic Insights into Cobweb Disease Resistance in *Agaricus bisporus*: A Comparative Analysis of Resistant and Susceptible Strains

Guohui Cheng[1,2], Xiaoya An[2], Yueting Dai[2*], Changtian Li[2*], Yu Li[1,2*]

[1] Department of Plant Protection, Shenyang Agricultural University, Shenyang 110866, China; chengguohui2016@163.com (G.-H.C.)

[2] Engineering Research Center of Chinese Ministry of Education for Edible and Medicinal Fungi, Jilin Agricultural University, Changchun 130118, China; anxiaoya2016@163.com (X.-Y.A.); daiyueting18@163.com (Y.-T.D.)

* Correspondence: lct@jlau.edu.cn (C.-T.L.); liyu@jlau.edu.cn (Y.L.)

Contents

Table S1. A list of *Agaricus bisporus* strains in this study and their specific characteristics. 1
Table S2. Assessment of DNA quality from the monokaryotic sample of *Agaricus bisporus* AB7. 2
Table S3. Statistics for Illumina PE150 sequencing data of *Agaricus bisporus* AB7 genome. 3
Table S4. Genomic survey of *Agaricus bisporus* strain AB7 using 15-mer based on Illumina PE150 sequencing data. 4
Table S5. Statistics of HiFi reads for *Agaricus bisporus* AB7 genome generated from subreads through the circular consensus sequence (CCS) algorithm. 5
Table S6. Statistics of BUSCO evaluation for *Agaricus bisporus* AB7 genome. 6
Table S7. Overall comparison of the genomes of *Agaricus bisporus* strains AB7 and AB58. 7
Table S8. Statistics of non-coding RNA within the genomes of *Agaricus bisporus* strains AB7 and AB58. 8
Table S9. Statistics of TEs within the genomes of *Agaricus bisporus* strains AB7 and AB58......... 9
Table S10. Statistics of TRs within the genomes of *Agaricus bisporus* strains AB7 and AB58. ... 10
Table S11. Statistics of SSRs within the genomes of *Agaricus bisporus* strains AB7 and AB58... 11
Table S12. Protein-coding gene annotation in *Agaricus bisporus* strains AB7 and AB58. 12
Table S13. Statistics of CAZyme families across ten genomes. 13
Table S14. Distribution of 84 GH classes across ten genomes. 14
Table S15. Distribution of 56 P450 classes across ten genomes. 16
Table S16. Statistics of secondary metabolite gene clusters across ten genomes. 18
Table S17. Characteristics of 11 NI-siderophore synthetase gene clusters across nine genomes. . 19
Table S18. Subcellular localization of proteins encoded by NI-siderophore synthetase genes. 22
Table S19. Statistics of resistance genes identified in the PRGdb database within the genomes of *Agaricus bisporus* strains AB7 and AB58. 23
Table S20. Distribution of resistance gene classes within the genomes of *Agaricus bisporus* strains AB7 and AB58. 24
Table S21. Statistics of transport factors identified in the Plant TFDB database within the genomes of *Agaricus bisporus* strains AB7 and AB58. 26
Table S22. The genetic composition of the pan-genome of strains AB7 and AB58. 27
Table S23. Statistics on the functional annotation of strain-specific genes in AB7 and AB58....... 28
Table S24. The software and databases used in this study. 29
Figure S1. Quality inspection of DNA from *Agaricus bisporus* strain AB7. 30
Figure S2. 15-mer frequency distribution and GC-sequencing depth correlation analysis of *Agaricus bisporus* strain AB7 genome. 31
Figure S3. HiFi read and gene length distributions of the *Agaricus bisporus* AB7 genome. 32
Figure S4. Visualized functional annotation of the *Agaricus bisporus* strain AB7 using KEGG, KOG, Nr, and GO databases. 33
Figure S5. Statistical analysis of the annotation results of ten genomes in the antiSMASH database. 34
Figure S6. Statistical analysis of TF families in *Agaricus bisporus* strains AB7 and AB58 predicted by Plant TFDB. 35

Table S1. A list of *Agaricus bisporus* strains in this study and their specific characteristics.

Preservation ID	Strain name	Original reference	Geographic origin	Strain category	Cap color	Collection year	Resistance level
CCMJ1013	AB7	As2796	Fujian, China	Cultivar	White	2015	HS
CCMJ1351	AB58	W2	Sichuan, China	Wild	Brown	2015	HR

Note: HS stands for high susceptibility to *Cladobotryum mycophilum* CM30, the causal agent of cobweb disease, while HR stands for high resistance to CM30.

Table S2. Assessment of DNA quality from the monokaryotic sample of *Agaricus bisporus* AB7.

Qubit (ng/μL) [a]	Qubit (μL) [b]	Qubit (μg) [c]	A260/280 [d]	A260/230 [e]	Nano (ng/μL) [f]	NC/QC [g]
156	67	10.452	1.854	1.958	214.946	1.38

Note: [a] DNA concentration measured by Qubit 2.0 fluorometer, with the unit of nanograms per microliter. [b] Volume of the sample measured by Qubit, with the unit of microliters. [c] Total amount of DNA calculated from the concentration measured by Qubit and the sample volume, with the unit of micrograms. [d] Ratio of absorbance at 260 nm to 280 nm of DNA measured by spectrophotometer. [e] Ratio of absorbance at 260 nm to 230 nm of DNA measured by spectrophotometer. [f] DNA concentration measured by NanoDrop 1000, with the unit of nanograms per microliter. [g] Ratio of the DNA concentration measured by NanoDrop to that measured by Qubit.

Table S3. Statistics for Illumina PE150 sequencing data of *Agaricus bisporus* AB7 genome.

Raw data (Mb)	Filtered reads (%)	Clean data (Mb)	Clean data GC (%)	Clean data Q20 (%)
2571	12.43	2251	45.77	96.91

Note: Raw data means the total amount of the initial sequencing data before filtering. Filtered reads means the ratio of filtered data to raw data. Clean data meaning the amount of valid data obtained after filtering. Clean data GC meaning the average GC content of valid data. Clean data Q20 meaning the Q20 value of valid data.

Table S4. Genomic survey of *Agaricus bisporus* strain AB7 using 15-mer based on Illumina PE150 sequencing data.

Number	Depth	Genome size (Mb)	Revised size (Mb)	Heterozygous (%)	Repeat (%)
1,676,875,220	51.53	32.54	31.8	0.03	25.68

Note: Number means the number of 15-mer extracted from reads for statistics analysis. Depth means the depth of 15-mer obtained from statistical analysis. Genome size means the estimated size of the genome. Revised size means the estimated size of genome after correction. Heterozygous means the estimated proportion of heterozygous regions in the genome. Repeat means the estimated proportion of genomic repeats.

Table S5. Statistics of HiFi reads for *Agaricus bisporus* AB7 genome generated from subreads through the circular consensus sequence (CCS) algorithm.

Num_seqs	Sum_length (bp)	Min_length (bp)	N50_length (bp)	Max_length (bp)
541,971	8,594,793,091	291	16,268	49,831

Note: Num_seqs means the count of HiFi reads generated from the sequencing process. Sum_len means the cumulative length of all HiFi reads. Min_len denotes the length of the shortest read. N50_len denotes the length of N50. Max_len denotes the length of the longest read.

Table S6. Statistics of BUSCO evaluation for *Agaricus bisporus* AB7 genome.

**BUSCO evaluation category**	**Number (#)**	**Percent (%)**
Complete BUSCOs (C)	716	94.5
Complete and single-copy BUSCOs (S)	710	93.7
Complete and duplicated BUSCOs (D)	6	0.8
Fragmented BUSCOs (F)	3	0.4
Missing BUSCOs (M)	39	5.1
Total BUSCO groups searched	758	100

Table S7. Overall comparison of the genomes of *Agaricus bisporus* strains AB7 and AB58.

**Item**	**AB7**	**AB58**
Coding (bp)	15,288,553 (50.63%)	12,762,102 (41.93%)
Non-coding (bp)	11,845,635 (39.23%)	14,635,810 (48.09%)
Repeat (bp)	3,062,379 (10.14%)	3,038,727 (9.98%)
Genome size (bp)	30,196,567	30,436,639

Table S8. Statistics of non-coding RNA within the genomes of *Agaricus bisporus* strains AB7 and AB58.

Type	Copy (w)		Total length (bp)		Coverage (%)	
	AB7	AB58	AB7	AB58	AB7	AB58
tRNA	173	201	14,142	16,246	0.04683	0.05338
rRNA	33	88	46,632	118,932	0.15443	0.39075
5s	9	21	989	2291	0.00328	0.00753
5.8s	8	20	1209	2294	0.004	0.00754
18s	8	22	14,422	36,892	0.04776	0.12121
28s	8	25	30,012	76,756	0.09939	0.25218
snoRNA	2	2	202	201	0.00067	0.00066
snRNA	8	9	1144	1248	0.00379	0.0041
miRNA	0	0	0	0	0	0
Total	216	300	62,120	13,6627	0.20572	0.44889

Table S9. Statistics of TEs within the genomes of *Agaricus bisporus* strains AB7 and AB58.

**Type**	**Copy (w)**		**Total length (bp)**		**Coverage (%)**	
	AB7	AB58	AB7	AB58	AB7	AB58
LINE	315	374	344,099	433,507	1.13953	1.42429
LTR	1776	1836	2,079,531	1,921,994	6.88665	6.31474
SINE	52	15	6913	1681	0.02289	0.00552
DNA	577	644	454,425	491,486	1.50489	1.61478
PLE	7	9	694	496	0.0023	0.00163
Unknown	21	17	1510	1433	0.005	0.00471
Total	2748	2895	2,887,172	2,850,597	9.56126	9.36568

Table S10. Statistics of TRs within the genomes of *Agaricus bisporus* strains AB7 and AB58.

**Type**	**Copy (w)**		**Total length (bp)**		**Coverage (%)**	
	AB7	AB58	AB7	AB58	AB7	AB58
Microsatellite	3226	3383	53,442	54,570	0.17698	0.17929
Minisatellite	1380	1458	75,771	88,115	0.25093	0.2895
Satellite	80	82	45,994	45,445	0.15232	0.14931
Total	4686	4923	175,207	188,130	0.58022	0.6181

Table S11. Statistics of SSRs within the genomes of *Agaricus bisporus* strains AB7 and AB58.

Type	AB7	AB58
Di-nucleotide (DNR)	1219	1223
Tri-nucleotide (TNR)	705	698
Tetra-nucleotide (TTNR)	13	22
Penta-nucleotide (PNR)	8	8
Hexan-nucleotide (HNR)	15	14
Total	1960	1965

Table S12. Protein-coding gene annotation in *Agaricus bisporus* strains AB7 and AB58.

Item	AB7	AB58
Nr (Non-Redundant Protein Database)	9708 (95.01%)	8413 (98.26%)
SwissProt	5028 (49.21%)	4257 (49.72%)
Pfam (Protein Families Database)	6119 (59.88%)	5531 (64.6%)
KEGG (Kyoto Encyclopedia of Genes and Genomes)	9771 (95.63%)	8249 (96.34%)
GO (Gene Ontology)	6119 (59.88%)	5199 (60.72%)
KOG (Clusters of Orthologous Groups for Eukaryotic)	4278 (41.87%)	3592 (41.95%)
CAZy (Carbohydrate-Active enZYmes Database)	1421 (13.91%)	1016 (11.87%)
P450 (CytochromeP450)	250 (2.45%)	176 (2.06%)
antiSMASH (antibiotics & Secondary Metabolite Analysis Shell)	198 (1.94%)	182 (2.13%)
PRGdb (Pathogen Receptor Genes Database)	364 (3.56%)	311 (3.63%)
Plant TFDB (Plant Transcription Factors Database)	134 (1.31%)	117 (1.37%)
Total	10,218	8562

Table S13. Statistics of CAZyme families across ten genomes.

Family	AB7	AB58	JB137-s8	H97	H119_p4	PC9	PC15	Lenedo1	Lenafn1	SCLS
AA	145	132	148	145	154	197	198	145	152	132
CBM	439	410	455	453	465	651	645	558	570	513
CE	215	86	119	109	268	111	111	114	111	111
GH	948	499	548	552	1054	653	667	693	712	676
GT	581	298	344	345	602	387	372	417	418	810
PL	94	16	17	16	125	34	34	20	20	88
Total	2422	1441	1631	1620	2668	2033	2027	1947	1983	2330

Table S14. Distribution of 84 GH classes across ten genomes.

Class	AB7	AB58	JB137-s8	H97	H119_p4	PC9	PC15	Lenedo1	Lenafn1	SCLS
GH0	13	14	15	15	13	20	16	28	24	12
GH1	37	15	16	17	34	17	18	15	17	14
GH10	2	2	2	2	2	5	4	5	6	3
GH100	61	0	2	1	77	0	0	0	0	6
GH101	2	2	2	2	2	1	2	1	1	1
GH104	0	0	0	0	0	1	1	0	0	0
GH105	4	6	4	5	4	5	5	8	8	3
GH106	0	0	0	0	0	0	0	3	1	1
GH109	2	2	2	2	2	3	4	4	3	2
GH11	2	2	2	2	2	2	2	2	2	0
GH115	2	2	2	2	2	1	1	1	1	2
GH117	0	0	0	0	0	0	0	1	1	0
GH12	2	2	2	2	2	2	2	4	4	2
GH125	2	1	1	1	1	1	1	1	1	1
GH127	0	0	0	1	1	1	0	2	2	1
GH128	3	3	2	2	3	4	5	7	7	3
GH13	16	14	16	16	18	17	18	24	23	18
GH131	3	3	3	2	3	5	4	4	4	3
GH132	1	1	1	1	1	1	1	0	0	3
GH133	1	1	1	1	0	1	1	0	0	1
GH134	3	2	3	3	3	2	5	2	2	5
GH135	0	0	0	0	0	0	1	1	1	4
GH140	0	0	0	0	0	0	0	0	0	1
GH141	0	0	0	0	0	0	1	1	1	0
GH145	1	0	1	1	1	1	2	1	1	1
GH146	2	2	2	2	2	1	2	1	1	0
GH15	0	1	1	1	1	2	2	1	1	2
GH152	12	14	14	17	16	22	24	19	20	23
GH154	1	1	1	1	1	1	1	2	2	1
GH16	87	28	32	33	91	35	35	41	41	20
GH162	1	1	1	1	1	1	1	0	0	0
GH17	90	8	9	8	97	5	5	9	9	48
GH18	58	59	63	64	59	74	69	69	68	49
GH19	5	5	5	5	5	6	8	4	4	4
GH2	6	4	5	7	6	6	7	9	9	8
GH20	5	5	6	6	6	4	4	5	7	5
GH23	18	13	18	19	20	24	21	20	25	18
GH24	2	2	1	1	2	3	3	0	0	0
GH25	4	4	4	4	3	2	2	1	2	0
GH26	0	0	0	0	0	0	0	1	1	0
GH27	4	4	4	4	4	4	7	7	7	6
GH28	139	14	18	17	173	21	22	33	37	70

GH29	4	2	3	3	3	1	1	5	4	2
GH3	39	35	40	39	40	44	42	38	42	27
GH30	3	3	3	3	3	5	5	5	6	2
GH31	7	9	9	9	9	15	15	11	13	20
GH32	8	6	9	10	7	5	6	6	7	9
GH33	0	0	0	0	0	1	1	1	1	0
GH35	3	3	3	3	3	6	7	5	5	6
GH36	24	23	25	25	26	30	31	31	30	29
GH37	21	20	21	21	21	23	24	25	29	20
GH38	92	4	5	5	109	6	5	5	6	51
GH39	0	0	0	0	0	1	1	1	1	1
GH4	3	3	3	3	3	6	6	6	7	2
GH43	33	31	34	32	36	44	43	40	41	32
GH44	1	1	1	1	1	1	1	0	0	0
GH45	0	1	1	1	1	3	3	3	3	1
GH47	14	13	16	15	17	16	19	15	15	11
GH5	32	37	35	36	35	37	37	37	42	24
GH51	1	1	1	1	2	3	3	3	3	5
GH53	1	1	1	1	1	1	1	2	3	1
GH55	8	8	8	8	8	8	8	10	9	11
GH6	9	8	10	10	11	11	10	16	14	10
GH62	0	0	0	0	0	1	1	0	0	0
GH63	2	2	2	2	2	2	2	2	2	2
GH65	3	2	3	3	3	3	3	3	3	2
GH7	1	1	1	1	1	12	16	4	4	1
GH71	6	7	6	7	6	10	10	13	13	6
GH72	1	1	1	1	1	1	1	1	1	5
GH74	1	1	1	1	1	3	3	1	1	1
GH75	0	0	0	0	0	4	5	3	3	3
GH76	3	3	2	2	3	3	4	11	8	8
GH78	10	11	15	13	16	13	15	9	10	8
GH79	6	5	6	6	5	7	7	8	8	3
GH81	0	0	0	0	0	0	0	0	0	1
GH84	1	0	1	1	1	1	1	1	1	1
GH85	2	2	2	2	2	2	2	2	2	3
GH88	1	1	1	1	1	2	1	2	2	6
GH9	9	8	9	9	9	11	11	11	11	5
GH92	4	5	5	5	5	6	6	6	6	8
GH93	2	2	2	2	2	1	1	4	5	4
GH94	0	0	0	0	0	1	0	1	1	1
GH95	1	1	1	1	1	1	1	8	5	2
GH99	1	1	1	1	1	1	1	1	1	1

Table S15. Distribution of 56 P450 classes across ten genomes.

Class	AB7	AB58	JB137-s8	H97	H119_p4	PC9	PC15	Lenedo1	Lenafn1	SCLS
CYP1	0	0	0	0	0	8	10	0	0	0
CYP102	1	2	1	1	1	2	1	4	3	6
CYP107	1	1	1	1	1	1	1	1	1	1
CYP108	0	0	0	0	0	0	0	0	0	1
CYP11	1	1	1	1	1	1	1	3	2	0
CYP116	0	0	0	0	0	0	0	0	0	0
CYP125	7	5	6	7	6	3	3	4	4	7
CYP140	0	0	0	0	0	0	0	0	0	0
CYP144	0	0	0	0	0	1	1	0	0	0
CYP146	1	1	1	1	1	0	0	0	0	0
CYP149	0	0	0	0	0	1	1	1	0	0
CYP158	0	0	0	0	0	0	0	0	0	1
CYP174	0	0	0	0	0	0	0	0	0	0
CYP2	1	1	1	1	1	13	12	1	2	0
CYP3	8	5	9	7	7	7	4	4	2	3
CYP35	25	1	11	0	22	0	0	0	0	6
CYP4	10	14	13	14	12	18	15	24	23	2
CYP46	1	1	0	0	0	0	0	0	0	0
CYP5	1	0	0	0	0	0	1	0	0	1
CYP503	0	0	0	0	0	1	1	0	0	0
CYP504	1	2	3	4	4	2	1	1	1	1
CYP505	4	4	4	4	4	3	4	9	7	6
CYP5060	0	0	0	0	0	0	0	0	0	0
CYP5080	0	0	0	0	0	0	0	0	0	2
CYP5099	0	0	0	0	0	0	0	0	0	0
CYP51	46	41	49	50	50	48	50	47	44	67
CYP510	0	0	0	0	0	0	0	6	5	0
CYP511	0	0	1	1	0	0	0	0	0	1
CYP512	12	9	13	13	14	6	6	8	8	3
CYP52	6	6	7	6	7	7	7	6	7	5
CYP526	0	0	0	0	0	0	0	0	0	0
CYP528	0	0	0	0	0	0	0	0	0	0
CYP5262	0	0	0	0	0	0	0	0	0	0
CYP5293	0	0	0	0	0	0	0	0	0	0
CYP53	9	8	9	9	9	7	7	8	9	4
CYP541	0	0	0	0	0	0	0	0	0	0
CYP542	0	0	0	0	0	0	0	0	0	0
CYP551	0	0	0	0	0	0	0	0	0	0
CYP561	0	0	0	0	0	0	0	0	0	0
CYP570	0	0	0	0	0	0	0	0	0	0
CYP578	1	1	1	1	1	1	1	1	1	2
CYP58	1	1	0	0	1	1	1	5	7	4

CYP584	0	0	0	0	0	0	0	0	0	0
CYP6	1	1	1	2	1	1	1	1	1	0
CYP61	1	1	1	1	1	4	2	1	1	1
CYP617	0	0	0	1	0	0	0	0	0	0
CYP620	0	0	0	0	0	0	0	0	0	0
CYP623	0	0	0	0	0	0	0	0	0	0
CYP625	0	0	0	0	0	0	0	0	0	0
CYP620	50	42	50	55	51	63	57	42	41	0
CYP628	0	0	0	0	0	0	0	0	0	0
CYP645	0	0	0	0	0	0	0	0	0	1
CYP65	0	0	0	0	0	0	1	3	2	3
CYP655	0	0	0	0	0	0	0	0	0	0
CYP660	0	0	0	0	0	0	0	0	0	0
CYP68	0	0	0	0	0	0	0	0	0	1
CYP682	0	0	0	0	0	0	0	0	0	0
CYP684	0	0	0	0	0	0	0	0	0	1
CYP7	0	0	0	0	0	1	1	0	0	0
CYP704	0	0	0	0	0	0	1	0	0	0
CYP705	0	0	0	0	0	0	0	1	1	0
CYP706	1	1	1	1	1	1	1	1	1	1
CYP709	0	0	0	0	0	0	0	1	2	0
CYP71	25	1	0	0	85	0	0	0	0	135
CYP715	4	0	0	0	4	0	0	2	1	0
CYP716	0	0	0	0	0	0	0	1	1	0
CYP749	3	3	4	4	4	2	3	4	4	1
CYP75	1	1	1	1	1	0	0	0	0	0
CYP76	2	2	2	2	2	10	8	2	4	0
CYP78	6	5	6	6	8	10	13	13	14	2
CYP79	0	0	0	0	1	0	0	0	0	0
CYP8	0	0	0	0	0	0	0	0	0	0
CYP81	8	7	8	7	7	5	5	7	8	1
CYP82	0	0	0	0	0	0	0	0	0	0
CYP83	9	7	5	5	6	3	5	4	4	2
CYP86	0	0	1	0	0	0	0	0	1	0
CYP88	0	0	0	0	0	1	1	0	0	0
CYP9	1	0	0	0	0	0	0	0	0	0
CYP94	1	1	1	1	1	2	2	0	0	0
CYP97	0	0	0	0	0	0	1	0	0	0

Table S16. Statistics of secondary metabolite gene clusters across ten genomes.

**Class**	**AB7**	**AB58**	**JB137-s8**	**H97**	**H119_p4**	**PC9**	**PC15**	**Lenedo1**	**Lenafn1**	**SCLS**
fungal-RiPP	0	0	0	0	0	0	0	1	1	1
fungal-RiPP-like	0	0	0	0	0	12	12	7	4	0
indole	1	2	2	2	2	0	0	0	0	0
NI-siderophore	1	1	1	1	1	1	1	2	2	0
NRP-metallophore	0	0	0	0	0	0	0	0	0	1
NRPS	0	0	0	0	0	1	1	2	2	1
NRPS-like	7	6	7	7	7	9	8	13	9	6
T1PKS	2	2	2	2	2	1	1	6	4	1
terpene	10	10	10	10	10	19	19	9	7	5
Total	21	21	22	22	22	43	42	40	29	15

Table S17. Characteristics of 11 NI-siderophore synthetase gene clusters across nine genomes.

Strain	Gene Name	Gene Location	Start	End	Length
AB7	A7.31.1_05156.1	Contig5	1140234	1142401	2168
AB7	A7.31.1_05157.1	Contig5	1143106	1144071	966
AB7	A7.31.1_05158.1	Contig5	1144353	1145326	974
AB7	A7.31.1_05159.1	Contig5	1151574	1153019	1446
AB7	A7.31.1_05162.1	Contig5	1159470	1165514	6045
AB7	A7.31.1_05163.1	Contig5	1167506	1168247	742
AB7	A7.31.1_05164.1	Contig5	1168519	1169080	562
AB7	A7.31.1_05165.1	Contig5	1169159	1171857	2699
AB7	A7.31.1_05160.1	Contig5	1153316	1155512	2197
AB7	A7.31.1_05161.1	Contig5	1156756	1158953	2198
AB58	Agaricus_bisporus_GLEAN_10005052	utg13	1150438	1151411	974
AB58	Agaricus_bisporus_GLEAN_10005053	utg13	1157720	1159166	1447
AB58	Agaricus_bisporus_GLEAN_10005056	utg13	1165530	1171660	6131
AB58	Agaricus_bisporus_GLEAN_10005057	utg13	1172349	1174405	2057
AB58	Agaricus_bisporus_GLEAN_10005058	utg13	1175317	1175956	640
AB58	Agaricus_bisporus_GLEAN_10005054	utg13	1159545	1161660	2116
AB58	Agaricus_bisporus_GLEAN_10005055	utg13	1163527	1165098	1572
JB137-s8	AGABI1DRAFT_40864	scaffold7	53834	55485	1652
JB137-s8	AGABI1DRAFT_107119	scaffold7	55484	57288	1805
JB137-s8	AGABI1DRAFT_114213	scaffold7	57437	58408	972
JB137-s8	AGABI1DRAFT_26292	scaffold7	64123	64504	382
JB137-s8	AGABI1DRAFT_59498	scaffold7	65359	66072	714
JB137-s8	AGABI1DRAFT_59499	scaffold7	66370	68566	2197
JB137-s8	AGABI1DRAFT_121041	scaffold7	69806	72003	2198
JB137-s8	AGABI1DRAFT_121043	scaffold7	72435	78567	6133
JB137-s8	AGABI1DRAFT_128884	scaffold7	79142	80338	1197
JB137-s8	AGABI1DRAFT_40995	scaffold7	80563	81304	742
JB137-s8	AGABI1DRAFT_41177	scaffold7	81580	82141	562
JB137-s8	AGABI1DRAFT_74928	scaffold7	82410	84673	2264
H97	AGABI2DRAFT_66879	scaffold3	1133685	1135335	1651
H97	AGABI2DRAFT_177227	scaffold3	1135334	1137138	1805
H97	AGABI2DRAFT_191229	scaffold3	1137287	1138260	974
H97	AGABI2DRAFT_66531	scaffold3	1143415	1144430	1016
H97	AGABI2DRAFT_218205	scaffold3	1144508	1145953	1446
H97	AGABI2DRAFT_218206	scaffold3	1146251	1148447	2197
H97	AGABI2DRAFT_183956	scaffold3	1149691	1151888	2198
H97	AGABI2DRAFT_201241	scaffold3	1152320	1158449	6130
H97	AGABI2DRAFT_116205	scaffold3	1159023	1160216	1194
H97	AGABI2DRAFT_191234	scaffold3	1160441	1161182	742
H97	AGABI2DRAFT_66278	scaffold3	1161454	1162015	562

H97	AGABI2DRAFT_201250	scaffold3	1162284	1164552	2269
H119_p4	Agabi119p4_5497	chromosome V	1225493	1227661	2169
H119_p4	Agabi119p4_5498	chromosome V	1227712	1229332	1621
H119_p4	Agabi119p4_5499	chromosome V	1229614	1230585	972
H119_p4	Agabi119p4_5500	chromosome V	1235667	1236729	1063
H119_p4	Agabi119p4_5501	chromosome V	1236807	1238252	1446
H119_p4	Agabi119p4_5502	chromosome V	1238550	1240746	2197
H119_p4	Agabi119p4_5503	chromosome V	1241986	1244183	2198
H119_p4	Agabi119p4_5504	chromosome V	1244615	1250747	6133
H119_p4	Agabi119p4_5505	chromosome V	1251322	1252518	1197
H119_p4	Agabi119p4_5506	chromosome V	1252743	1254321	1579
H119_p4	Agabi119p4_5507	chromosome V	1254400	1256853	2454
PC15	PC9H_009557	scaffold7	1061774	1062273	500
PC15	PC9H_009558	scaffold7	1062606	1065319	2714
PC15	PC9H_009559	scaffold7	1066217	1072641	6425
PC15	PC9H_009560	scaffold7	1073276	1075494	2219
PC15	PC9H_009561	scaffold7	1076969	1078932	1964
PC15	PC9H_009562	scaffold7	1079311	1080948	1638
PC15	PC9H_009563	scaffold7	1081028	1081478	451
PC15	PC9H_009564	scaffold7	1083438	1085853	2416
PC15	PC9H_009565	scaffold7	1088339	1089291	953
PC15	PC9H_009566	scaffold7	1090935	1092694	1760
PC15_2	PLEOSDRAFT_1107430	scaffold8	978834	979371	538
PC15_2	PLEOSDRAFT_1067328	scaffold8	979704	982417	2714
PC15_2	PLEOSDRAFT_1046717	scaffold8	983315	989739	6425
PC15_2	PLEOSDRAFT_1078605	scaffold8	990395	993111	2717
PC15_2	PLEOSDRAFT_161454	scaffold8	994067	996169	2103
PC15_2	PLEOSDRAFT_1113819	scaffold8	996414	998051	1638
PC15_2	PLEOSDRAFT_161451	scaffold8	1000568	1002983	2416
PC15_2	PLEOSDRAFT_1097745	scaffold8	1005470	1006423	954
PC15_2	PLEOSDRAFT_1085679	scaffold8	1008192	1009951	1760
Lenedo1	C8R40DRAFT_1096815	scaffold11	322216	324701	2486
Lenedo1	C8R40DRAFT_1159866	scaffold11	325148	326283	1136
Lenedo1	C8R40DRAFT_85254	scaffold11	326437	327258	822
Lenedo1	C8R40DRAFT_1041358	scaffold11	327953	328438	486
Lenedo1	C8R40DRAFT_85305	scaffold11	331363	333883	2521
Lenedo1	C8R40DRAFT_1271692	scaffold11	335842	338039	2198
Lenedo1	C8R40DRAFT_1041313	scaffold11	339062	345507	6446
Lenedo1	C8R40DRAFT_1183075	scaffold11	346056	350065	4010
Lenedo1	C8R40DRAFT_1074702	scaffold11	3237	5747	2511
Lenedo1	C8R40DRAFT_1061644	scaffold67	6697	10529	3833
Lenedo1	C8R40DRAFT_1134302	scaffold67	11163	12288	1126
Lenedo1	C8R40DRAFT_1163920	scaffold67	12938	13829	892

Lenedo1	C8R40DRAFT_819471	scaffold67	14481	17432	2952
Lenedo1	C8R40DRAFT_1061709	scaffold67	18059	19611	1553
Lenafn1	C8J55DRAFT_251472	scaffold5	239353	241824	2472
Lenafn1	C8J55DRAFT_468723	scaffold5	242262	243401	1140
Lenafn1	C8J55DRAFT_502575	scaffold5	243558	244374	817
Lenafn1	C8J55DRAFT_502577	scaffold5	244598	244888	291
Lenafn1	C8J55DRAFT_251509	scaffold5	245097	248180	3084
Lenafn1	C8J55DRAFT_419735	scaffold5	248495	250890	2396
Lenafn1	C8J55DRAFT_540579	scaffold5	252919	255116	2198
Lenafn1	C8J55DRAFT_468735	scaffold5	256364	262579	6216
Lenafn1	C8J55DRAFT_449147	scaffold5	263121	267130	4010
Lenafn1	C8J55DRAFT_441622	scaffold5	53574	54906	1333
Lenafn1	C8J55DRAFT_609787	scaffold54	56067	56637	571
Lenafn1	C8J55DRAFT_529296	scaffold54	56800	57268	469
Lenafn1	C8J55DRAFT_264083	scaffold54	60414	60940	527
Lenafn1	C8J55DRAFT_264111	scaffold54	62718	65212	2495
Lenafn1	C8J55DRAFT_493640	scaffold54	77457	78498	1042

Table S18. Subcellular localization of proteins encoded by NI-siderophore synthetase genes.

**Strain**	**Gene Name**	**Predicting Subcellular Localization**
AB7	A7.31.1_05160	Extracell; Cytoplasm
AB7	A7.31.1_05161	Extracell
AB58	Agaricus_bisporus_GLEAN_10005054	Extracell; Cell membrane; Cytoplasm; Endoplasmic reticulum; Nucleus
AB58	Agaricus_bisporus_GLEAN_10005055	Cytoplasm; Nucleus
JB137-s8	AGABI1DRAFT_59499	Extracell
JB137-s8	AGABI1DRAFT_121041	Extracell
H97	AGABI2DRAFT_218206	Extracell; Cytoplasm
H97	AGABI2DRAFT_183956	Extracell
H119_p4	Agabi119p4_5502	Extracell
H119_p4	Agabi119p4_5503	Extracell
PC15	PC9H_009560	Extracell
PC15	PC9H_00956	Extracell
PC15_2	PLEOSDRAFT_1078605	Extracell
PC15_2	PLEOSDRAFT_161454	Extracell
Lenedo1	C8R40DRAFT_1271692	Extracell; Endoplasmic reticulum
Lenedo1	C8R40DRAFT_1074702	Cytoplasm; Endoplasmic reticulum; Nucleus
Lenafn1	C8J55DRAFT_540579	Extracell; Endoplasmic reticulum
Lenafn1	C8J55DRAFT_264111	Cytoplasm; Endoplasmic reticulum; Nucleus

Table S19. Statistics of resistance genes identified in the PRGdb database within the genomes of *Agaricus bisporus* strains AB7 and AB58.

**Class**	**AB7**	**AB58**
CN	2	0
CNL	239	184
N	9	5
NL	30	23
TNL	64	46
RLK	939	897
RLP	54	49
T	15	13
TN	1	2
KIN	104	101
Others	845	793

Table S20. Distribution of resistance gene classes within the genomes of *Agaricus bisporus* strains AB7 and AB58.

Class	Gene	AB7	AB58	Class	Gene	AB7	AB58
CN	*pb1*	2	0	NL	Pi54	1	1
CNL	*Bs2*	2	2	TNL	Bs4	7	5
CNL	*MLA10*	4	3	TNL	P2	2	2
CNL	*Gpa2*	8	8	TNL	Gro1.4	3	3
CNL	*Hero*	0	1	TNL	N	7	5
CNL	*Mi1.2*	3	1	TNL	L6	2	1
CNL	*Prf*	2	2	TNL	M	4	2
CNL	*R1*	7	8	TNL	RPP5	2	1
CNL	*Rpi-blb1*	6	5	TNL	Rps4	4	2
CNL	*Dm3(RGC2B)*	2	1	TNL	RPP1	9	6
CNL	*HRT*	1	1	TNL	RPP4	3	4
CNL	*Pi-ta*	2	3	TNL	RRS1	3	2
CNL	*RCY1*	2	1	TNL	RY-1	9	6
CNL	*Rpi-blb2*	1	1	TNL	KR1	1	1
CNL	*RPM1*	5	4	TNL	RAC1	2	1
CNL	*RPP8*	1	0	TNL	L,L1-L11	3	2
CNL	*Rps2*	8	7	TNL	P,P1-4	2	2
CNL	*RPS5*	9	9	TNL	FOM1	1	1
CNL	*Rx*	7	8	RLK	Cf-2	9	9
CNL	*PIB*	7	2	RLK	Cf-4	6	5
CNL	*XA1*	0	2	RLK	Cf4A	8	7
CNL	*Rx2*	7	6	RLK	Cf-5	8	8
CNL	*Tm-2a*	5	2	RLK	FLS2	106	102
CNL	*Tm-2*	6	2	RLK	EFR	108	105
CNL	*MLA1*	2	3	RLK	PEPR1	114	106
CNL	*Mla6*	4	3	RLK	ER-Erecta	105	99
CNL	*Mla12*	3	3	RLK	xa21	94	91
CNL	*MLA13*	4	3	RLK	Xa26	102	94
CNL	*Pi36*	2	3	RLK	Serk3A	91	88
CNL	*Rp1-D*	2	1	RLK	Serk3B	91	88
CNL	*Pm3*	3	4	RLK	BAK1	97	95
CNL	*Lr10*	3	4	RLP	LeEIX1	10	7
CNL	*Pl8*	9	3	RLP	LeEIX2	10	10
CNL	*SSI4*	3	3	RLP	Cf-9	6	7
CNL	*Pi9*	1	2	RLP	Cf9B	6	5
CNL	*Piz-t*	2	2	RLP	Ve1	9	6
CNL	*Pi2*	2	2	RLP	Ve2	7	7
CNL	*Cre1*	2	0	RLP	RPP27	6	7
CNL	*Pikm1-TS*	8	6	T	RPP2A	6	7
CNL	*Pikm2-TS*	1	0	T	prv	9	6
CNL	*Rdg2a*	1	1	TN	RLM3	1	2

CNL	*Pid3*	5	5	KIN	Pto	104	101
CNL	*Pi5-1*	4	2	Others	Asc-1	2	2
CNL	*Pi5-2*	11	4	Others	Bs3	13	10
CNL	*Pit*	2	1	Others	Bs3-E	13	10
CNL	*Pikp-2*	9	6	Others	PGIP	3	2
CNL	*FOM-2*	2	0	Others	RPG1	98	92
CNL	*Lr21*	4	3	Others	At1	2	2
CNL	*Lr1*	1	2	Others	At2	2	3
CNL	*VAT*	6	6	Others	Hm1	7	5
CNL	*Sr33*	4	2	Others	RFO1	103	98
CNL	*Sr35*	5	2	Others	Hm2	4	4
CNL	*Cre3*	2	0	Others	RTM2	1	1
CNL	*Yr10*	4	2	Others	Xa5	1	1
CNL	*Ph-3*	4	1	Others	Pid2	107	104
CNL	*RB*	6	5	Others	Lr34	45	37
CNL	*Pia*	1	1	Others	Hcr9-4E	8	7
CNL	*Pi37*	3	3	Others	Fen	105	100
CNL	*Rpr1*	16	15	Others	Pti1	104	98
CNL	*L3*	3	1	Others	PBS1	98	98
CNL	*RGA5*	0	1	Others	Ty-1	8	6
N	*RGA2*	2	1	Others	Ty-3	8	6
N	*Rxo1*	6	4	Others	Ty-5	1	1
N	*NRC1*	1	0	Others	Pvr2	4	4
NL	*I-2*	2	1	Others	ADR1	1	2
NL	*R3a*	8	4	Others	Yr36	107	100
NL	*Rps1-k-2*	6	5				
NL	*Rps1-k-1*	6	6				
NL	*CHS3/DAR4*	7	6				

Table S21. Statistics of transport factors identified in the Plant TFDB database within the genomes of *Agaricus bisporus* strains AB7 and AB58.

**Family**	**AB7**	**AB58**
bHLH	11	11
bZIP	6	6
C2H2	53	44
C3H	21	15
GATA	10	11
HB-other	5	2
HD-ZIP	4	4
HSF	6	6
M-type_MADS	2	2
MYB	6	5
MYB_related	3	4
NF-YA	1	1
NF-YB	3	2
NF-YC	2	3
YABBY	1	1

Table S22. The genetic composition of the pan-genome of strains AB7 and AB58.

	**AB7**	**AB58**
All gene	10,218	8562
Pan gene	5807	5692
Core gene	2327	2987
Dispensable gene	3480	2705
Specific gene	4130	2803

Table S23. Statistics on the functional annotation of strain-specific genes in AB7 and AB58.

Sample ID	KEGG	KOG	GO	Pfam
AB7_speci	3365	444	2125	2125
AB58_speci	2255	302	1362	1362

Table S24. The software and databases used in this study.

Software and Databases	Website
CCS	https://github.com/pacificbiosciences/ccs
Hifiasm	https://github.com/chhylp123/hifiasm
GeneMarkHMM	https://github.com/gatech-genemark/GeneMarkHmmEukaryotic3
FGENESH	https://www.softberry.com/
Augustus	https://github.com/Gaius-Augustus/Augustus
SNAP	https://github.com/snap-stanford/snap
GlimmerHMM	http://ccb.jhu.edu/software/glimmerhmm/
GeneWise	https://www.ebi.ac.uk/Tools/psa/genewise/
Trinity	https://github.com/trinityrnaseq/trinityrnaseq
PASA	https://github.com/PASApipeline/PASApipeline
EVM	https://github.com/EVidenceModeler/EVidenceModeler
RepeatModeler2	https://github.com/Dfam-consortium/RepeatModeler
RepeatMasker	https://github.com/rmhubley/RepeatMasker
MicroSAtellite	https://mybiosoftware.com/misa-microsatellite-identification-tool.html
TRF	https://tandem.bu.edu/trf/trf.html
tRNAscan-SE	https://github.com/UCSC-LoweLab/tRNAscan-SE
Barrnap	https://github.com/tseemann/barrnap
TBtool-II	https://github.com/CJ-Chen/TBtools-II/releases
MapChart	https://www.wur.nl/en/show/Mapchart.htm
MUMmer	https://github.com/chienchi/MUMmer
LASTZ	https://github.com/lastz/lastz
CD-HIT	https://github.com/weizhongli/cdhit
UniRef90	https://www.uniprot.org/
Rfam	https://github.com/Rfam/rfam-production
MEME	https://meme-suite.org/meme/tools/meme
PredictProtein	https://predictprotein.org/
SWISS-MODEL	https://swissmodel.expasy.org/
NR	https://www.ncbi.nlm.nih.gov/
Swiss-Prot	https://www.uniprot.org/
Pfam	https://www.ebi.ac.uk/interpro/
KEGG	https://www.kegg.jp/
GO	http://www.geneontology.org
KOG	http://eggnogdb.embl.de/
CAZy	http://www.cazy.org
Fungal Cytochrome P450	http://p450.riceblast.snu.ac.kr/
Fungal antiSMASH	https://fungismash.secondarymetabolites.org/
Plant TFDB	https://planttfdb.gao-lab.org/
PRGdb	http://prgdb.org/prgdb4/

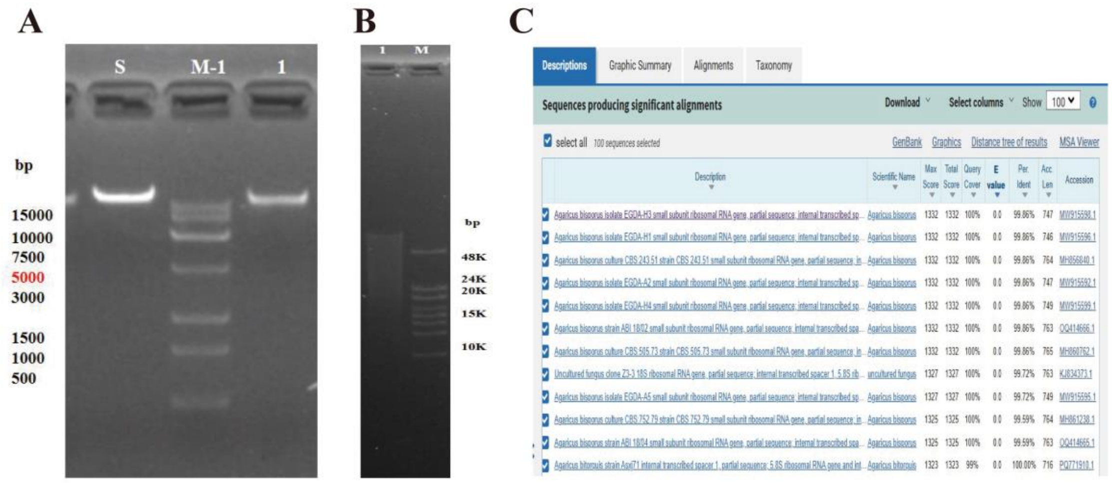

Figure S1. Quality inspection of DNA from *Agaricus bisporus* strain AB7.

(A) 1% agarose gel electrophoresis of strain AB7 genomic DNA at 180V for 20 min. M-1 is Trans 15k plus (2 μl loaded), S is the standard sample (5 μl, 10 ng/μl), and 1 is the sample (1 μl, 5-fold diluted of the original solution). (B) 0.8% pulsed-field agarose gel electrophoresis of strain AB7 genomic DNA at 5-80K for 17 h. M is 48Kb GeneRuler High Range DNA Ladder, and 1 is the sample (1 μl, 5-fold diluted of the original solution). (C) ITS sequence alignment of strain AB7 against NCBI nr database.

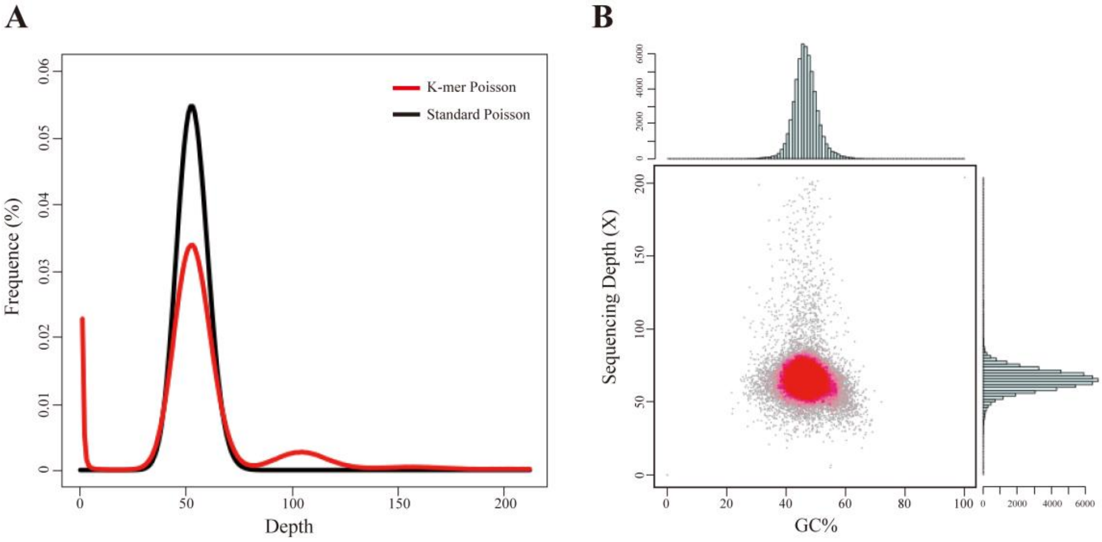

Figure S2. 15-mer frequency distribution and GC-sequencing depth correlation analysis of *Agaricus bisporus* strain AB7 genome.

(A) Distribution of 15-mer frequency for strain AB7. The x-axis indicates the k-mer depth, and the y-axis indicates the relative frequency (frequency at each depth/total frequency). The red curve represents the 15-mer depth distribution of the sequencing data, while the black curve represents the closest standard Poisson distribution. (B) Correlation analysis of GC content and sequencing depth for strain AB7. The x-axis indicates the GC content, and the y-axis indicates the sequencing depth. The distribution of sequencing depth is presented on the right, and the distribution of GC content on the top.

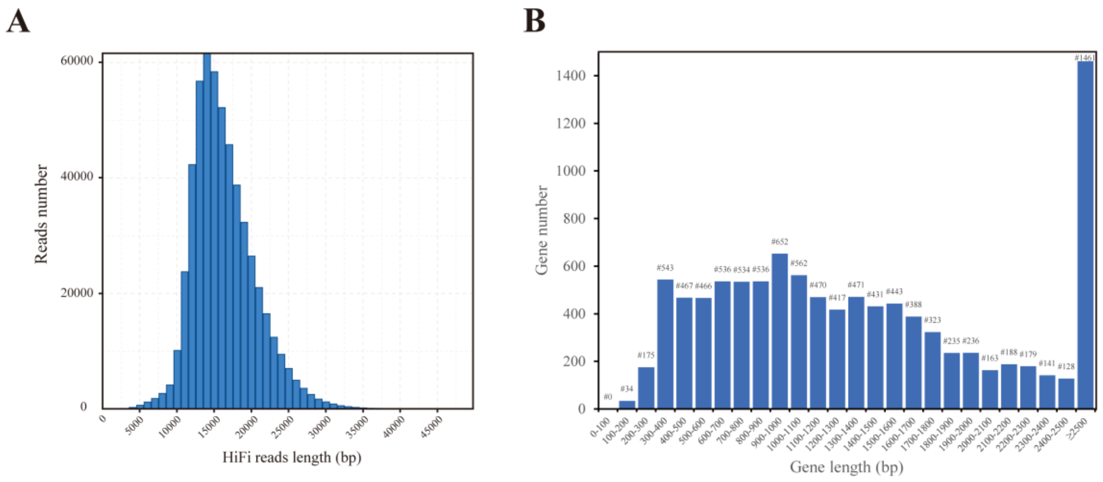

Figure S3. HiFi read and gene length distributions of the *Agaricus bisporus* AB7 genome.

(A) Length distribution of HiFi reads for strain AB7. The x-axis represents the sequencing read length, and the left y-axis represents the number of reads per read length. (B) Length distribution of genes for strain AB7. The x-axis represents the gene length, and the y-axis represents the number of genes at each length.

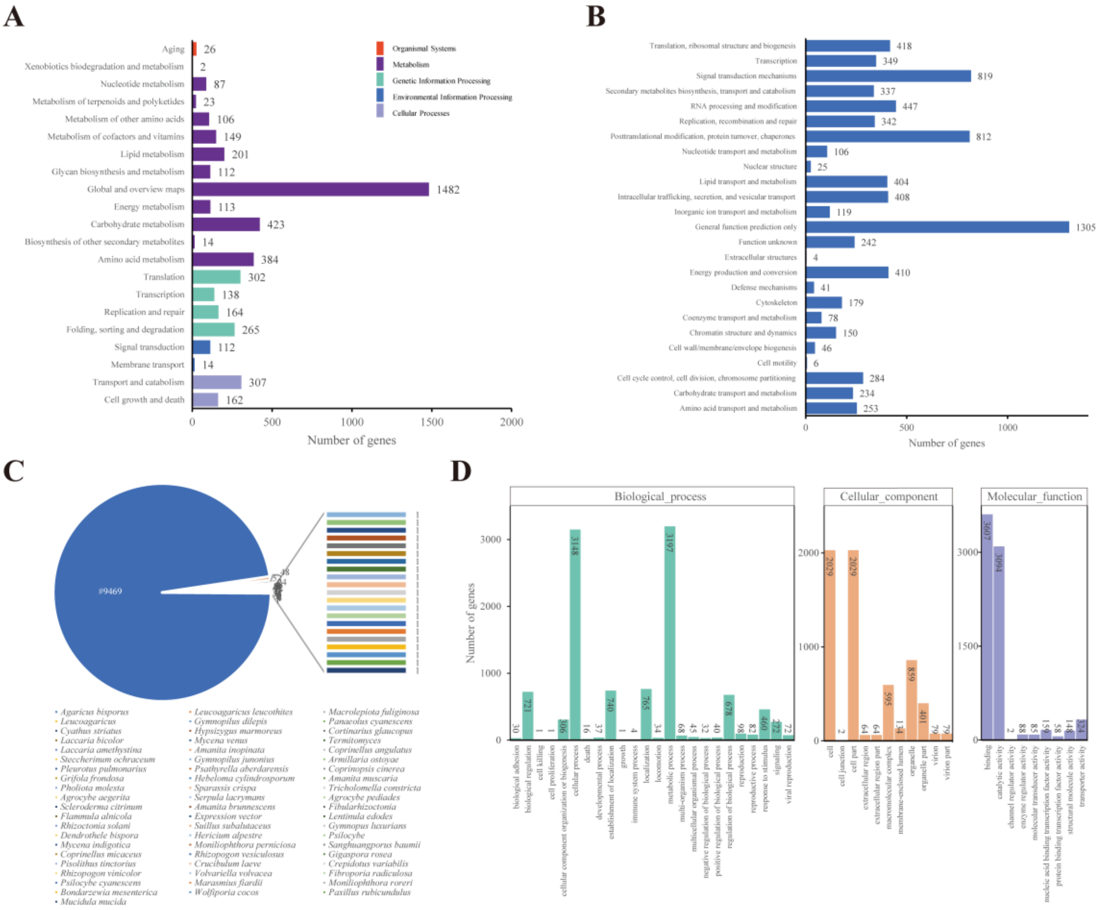

Figure S4. Visualized functional annotation of the *Agaricus bisporus* strain AB7 using KEGG, KOG, Nr, and GO databases.

(A) KEGG pathway classification chart of strain AB7. (B) KOG classification chart of strain AB7. (C) Species distribution map of the alignment results of strain AB7 genome sequences to the Nr database. (D) GO classification map of strain AB7.

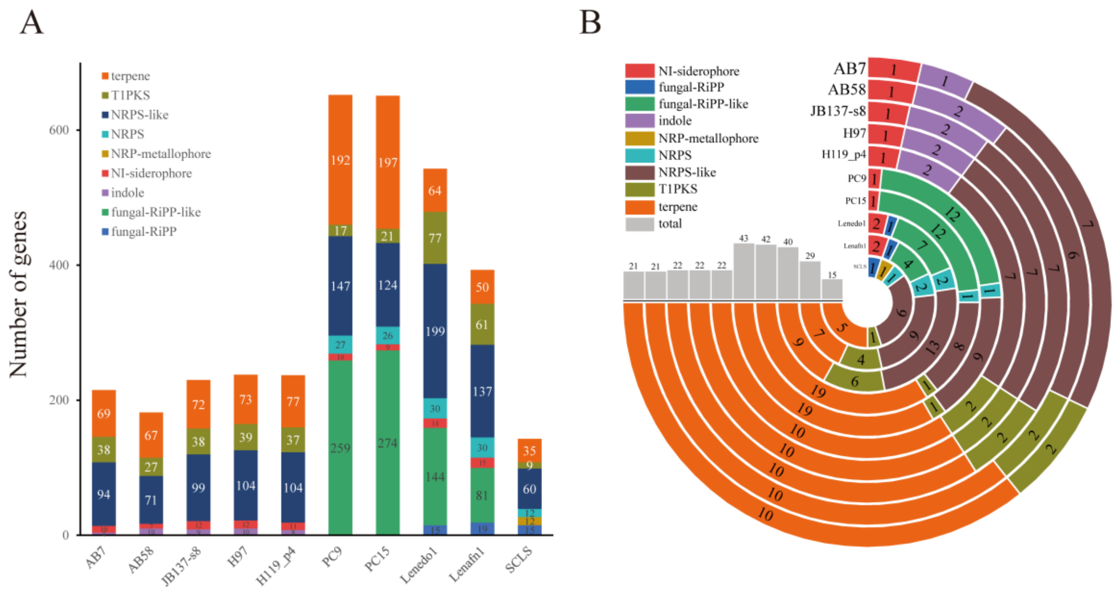

Figure S5. Statistical analysis of the annotation results of ten genomes in the antiSMASH database.

(A) Statistical analysis of the genes annotated across ten genomes. (B) Statistical analysis of the secondary metabolite gene clusters across ten genomes.

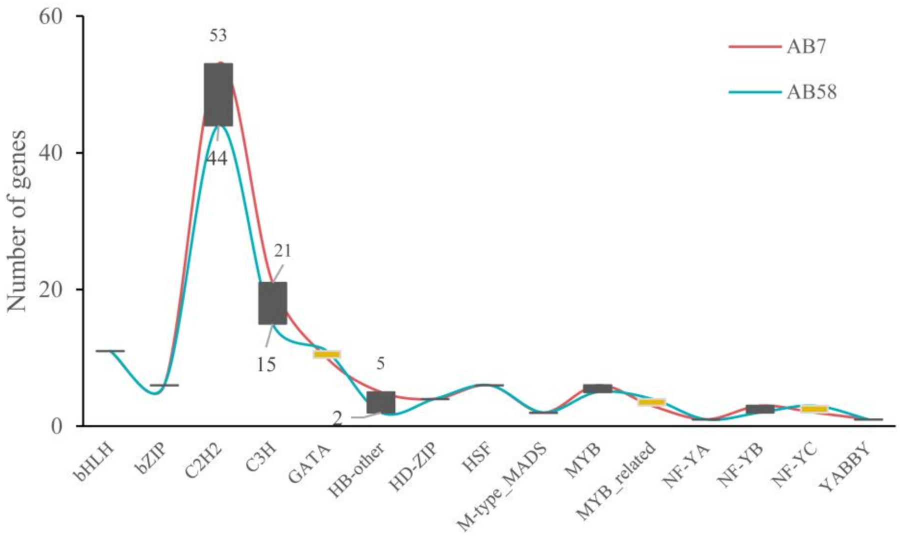

Figure S6. Statistical analysis of TF families in *Agaricus bisporus* strains AB7 and AB58 predicted by Plant TFDB.

The bar graphs quantify the difference in the number of annotated genes between the two strains. Black bars indicate that strain AB7 has more genes than AB58 within a given TF family, while gold bars indicate the opposite.
